# Disease-specific variant pathogenicity prediction significantly improves variant interpretation in inherited cardiac conditions

**DOI:** 10.1038/s41436-020-00972-3

**Published:** 2020-10-13

**Authors:** Xiaolei Zhang, Roddy Walsh, Nicola Whiffin, Rachel Buchan, William Midwinter, Alicja Wilk, Risha Govind, Nicholas Li, Mian Ahmad, Francesco Mazzarotto, Angharad Roberts, Pantazis I. Theotokis, Erica Mazaika, Mona Allouba, Antonio de Marvao, Chee Jian Pua, Sharlene M. Day, Euan Ashley, Steven D. Colan, Michelle Michels, Alexandre C. Pereira, Daniel Jacoby, Carolyn Y. Ho, Iacopo Olivotto, Gunnar T. Gunnarsson, John L. Jefferies, Chris Semsarian, Jodie Ingles, Declan P. O’Regan, Yasmine Aguib, Magdi H. Yacoub, Stuart A. Cook, Paul J. R. Barton, Leonardo Bottolo, James S. Ware

**Affiliations:** 1 National Heart and Lung Institute, Imperial College London, London, United Kingdom; 2 Cardiovascular Research Centre, Royal Brompton and Harefield NHS, Foundation Trust London, London, United Kingdom; 3 MRC London Institute of Medical Sciences, Imperial College London, London, United Kingdom; 4 Cardiomyopathy Unit, Careggi University Hospital, Florence, Italy; 5 Department of Clinical and Experimental Medicine, University of Florence, Florence, Italy; 6 Aswan Heart Centre, Magdi Yacoub Heart Foundation, Aswan, Egypt; 7 National Heart Centre, Singapore, Singapore; 8 Division of Cardiovascular Medicine and Penn Cardiovascular Institute, Perelman School of Medicine, University of Pennsylvania, Philadelphia, USA; 9 Division of Cardiovascular Medicine, Stanford University Medical Center, Stanford, CA USA; 10 Department of Cardiology, Boston Children’s Hospital, Boston, MA USA; 11 Department of Cardiology, Thoraxcenter, Erasmus MC Rotterdam, Rotterdam, Netherlands; 12 Heart Institute (InCor), University of Sao Paulo Medical School, Sao Paulo, Brazil; 13 Department of Internal Medicine, Yale University, New Haven, CT USA; 14 Cardiovascular Division, Brigham and Women’s Hospital, Boston, MA USA; 15 Faculty of Medicine, University of Iceland, Akureyri, Iceland; 16 The Cardiovascular Institute, University of Tennessee, Memphis, TN USA; 17 Centenary Institute, The University of Sydney, Sydney, Australia; 18 Department of Cardiology, Royal Prince Alfred Hospital, Sydney, Australia; 19 Duke-National University of Singapore, Singapore, Singapore; 20 Department of Medical Genetics, University of Cambridge, Cambridge, United Kingdom; 21 Alan Turing Institute, London, United Kingdom; 22 MRC Biostatistics Unit, University of Cambridge, Cambridge, United Kingdom

**Keywords:** pathogenicity prediction, missense variant interpretation, cardiomyopathy, long QT syndrome, Brugada syndrome

## Abstract

**Purpose:**

Accurate discrimination of benign and pathogenic rare variation remains a priority for clinical genome interpretation. State-of-the-art machine learning variant prioritization tools are imprecise and ignore important parameters defining gene–disease relationships, e.g., distinct consequences of gain-of-function versus loss-of-function variants. We hypothesized that incorporating disease-specific information would improve tool performance.

**Methods:**

We developed a disease-specific variant classifier, CardioBoost, that estimates the probability of pathogenicity for rare missense variants in inherited cardiomyopathies and arrhythmias. We assessed CardioBoost’s ability to discriminate known pathogenic from benign variants, prioritize disease-associated variants, and stratify patient outcomes.

**Results:**

CardioBoost has high global discrimination accuracy (precision recall area under the curve [AUC] 0.91 for cardiomyopathies; 0.96 for arrhythmias), outperforming existing tools (4–24% improvement). CardioBoost obtains excellent accuracy (cardiomyopathies 90.2%; arrhythmias 91.9%) for variants classified with >90% confidence, and increases the proportion of variants classified with high confidence more than twofold compared with existing tools. Variants classified as disease-causing are associated with both disease status and clinical severity, including a 21% increased risk (95% confidence interval [CI] 11–29%) of severe adverse outcomes by age 60 in patients with hypertrophic cardiomyopathy.

**Conclusions:**

A disease-specific variant classifier outperforms state-of-the-art genome-wide tools for rare missense variants in inherited cardiac conditions (https://www.cardiodb.org/cardioboost/), highlighting broad opportunities for improved pathogenicity prediction through disease specificity.

## INTRODUCTION

The accurate prediction of the effect of a previously unseen genetic variant on disease risk is an unmet need in clinical genetics. According to guidelines developed by the American College of Medical Genetics and Genomics/Association for Molecular Pathology (ACMG/AMP),^1^ computational prediction of variant pathogenicity is integrated as one line of supporting evidence to assess the clinical significance of genetic variation. Several tools have been developed to predict the effects of rare variants given multiple functional annotations to derive scores describing the likelihood of pathogenicity.^2–6^

While existing genome-wide tools learn from large-scale data over the entire genome, they might also compromise the prediction accuracy for specific sets of genes and diseases^7^ in the following ways. First, variation in a single gene can cause distinct phenotypes via different allelic mechanisms. Genome-wide tools that classify variants as deleterious or not, without reference to a specific disease or mechanism, may not perform as well as those that separate gene–disease relations since, for example, they do not distinguish between gain- and loss-of-function variants. Second, genome-wide classification tools may not benefit from specific lines of evidence only available for a subset of well-characterized genes or diseases. We have previously shown^8^ that the addition of gene- and disease-specific evidence into a classification model improves variant interpretation in inherited cardiac diseases. Finally, most genome-wide prediction tools are reported to have low specificity.^1^

Furthermore, the measures used in the evaluation of existing genome-wide tools are not always well defined or the most clinically relevant. The performance of variant classification is routinely evaluated using conventional classification performance measures such as the receiver operating characteristic (ROC) curve, which assesses diagnostic performance across a range of discrimination thresholds, or metrics such as sensitivity and specificity derived with a single and specified threshold. We argue that these measures should be tailored to the specific application at hand. In particular, it is necessary to consider the relative cost of decisions based on the type I and type II errors in any specific application, as different contexts may favor the control of type I error (limiting false positive assertions) or type II error (limiting false negative assertions). For example, when classifying a variant for predictive genetic testing, control of the type I error is usually prioritized: familial cascade testing on a variant falsely reported as pathogenic can be extremely harmful.^9^ Conversely, if considering whether to offer therapy effective in a subgroup of patients with a particular molecular etiology (e.g., sulfonylureas in monogenic diabetes^10^), one might prioritize control of type II error, to identify all those who might benefit from targeted treatment when the benefits outweigh the side effects. Most current genome-wide in silico variant classifiers favor sensitivity over control of the type I error, and thus overpredict disease-causing variants.^1^ The inappropriate use of performance measures not only affects the construction of the best classifier, but also the evaluation of its utility in clinical applications.

To address the disadvantages of genome-wide classification tools, we sought to develop an accurate variant classifier considering gene–disease relations by taking inherited cardiac conditions (ICCs) as examples. The resulting disease-specific variant classification tool, CardioBoost, includes two disease-specific variant classifiers for two groups of closely related syndromes: one classifier for familial cardiomyopathies (CM) that include hypertrophic cardiomyopathy (HCM) and dilated cardiomyopathy (DCM), and the other for inherited arrhythmia syndromes (IAS) that include long QT syndrome (LQTS) and Brugada syndrome.

While optimally it may be desirable to train a specific model for every gene–disease pair, this is not feasible due to current limitations in the number of variants with well-characterized disease consequences. Moreover, we have previously demonstrated benefit from jointly fitting some parameters across closely related genes or diseases.^8^ We therefore constructed models that aggregate genes of closely related syndromes, hypothesizing that these disease-specific models are biologically plausible since the computational evidence to interpret variant effect is more likely transferable within closely related syndromes.

Trained on well-curated disease-specific data, CardioBoost integrates multiple variant annotations and pathogenicity scores obtained from previously computational tools, and predicts the probability that rare missense variants are disease-causing for monogenic inherited cardiac conditions, based on the Adaptive Boosting (AdaBoost) algorithm.^11^ Our tool has improved performances over state-of-the-art genome-wide tools in several distinct tasks including discrimination of disease-causing from benign variants, prioritization of variants highly associated with disease, and prioritization of variations that stratify clinical outcomes.

## MATERIALS AND METHODS

A full description of data collection, model development, and validation is given in the Supplementary Methods. In brief, we constructed two classifiers, one for inherited cardiomyopathies, and one for inherited arrhythmia syndromes, to output the estimated probability of pathogenicity for rare missense variants in genes robustly associated with these diseases. Throughout this article, we reserve the standard terms “pathogenic” (P) or “likely pathogenic” (LP) recommended in the ACMG/AMP guidelines for variant assertions from ClinVar, or for variants that have been evaluated using the full ACMG/AMP framework. We use “disease-causing”/“likely disease-causing” to indicate pathogenicity predictions from in silico tools including CardioBoost and benchmarked tools.

The CM classifier is applicable for 16 genes associated with hypertrophic and dilated cardiomyopathies. To obtain training and test sets, we collected 356 unique rare (gnomAD minor allele frequency <0.1%) missense variants in established cardiomyopathy-associated genes (Table S1) identified in 9007 individuals with a clinical diagnosis of CM, and interpreted as pathogenic or likely pathogenic. For the inherited arrhythmia classifier, we consider genes associated with long QT syndrome and Brugada syndrome. To maximize the size and diversity of the training data, we used ClinVar and only included variants with no conflicting interpretation (conflicting: P/LP vs. benign/likely benign [B/LB]; P/LP vs. variant of uncertain significance [VUS]; B/LB vs. VUS). Two hundred fifty-two unique rare missense variants reported to be P or LP with no conflicting interpretations (B or LB) in established arrhythmia-associated genes (Table S2) were collected from the ClinVar database.^12^ As a benign variant set, 302 unique rare missense variants in cardiomyopathy genes, and 237 unique rare missense variants in arrhythmia genes, were collected from the targeted sequencing of 2090 healthy volunteers. Since these volunteers have no family history of ICCs and confirmed without ICCs on electrocardiogram (ECG) or cardiac magnetic resonance image (MRI), this cohort provides a lower disease prevalence than a general population, thus the rare missense variants carried by them should be considered as highly likely benign to inherited cardiac conditions. To avoid overfitting, for each condition the data set were randomly split, with two-thirds used for training and one-third reserved as a holdout test set (Tables S3–S5). For cardiomyopathies, 440 and 326 variants are used for training and testing, respectively. For arrhythmias, 218 and 166 variants are used for training and testing, respectively.

**Fig. 1:**
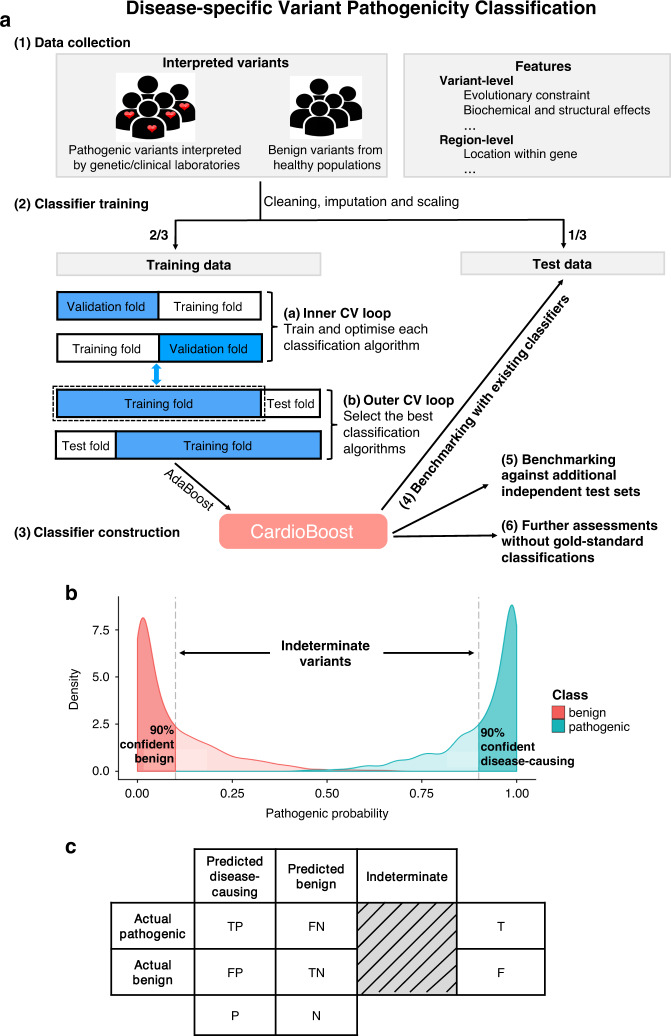
Training and testing of CardioBoost, and definition of high-confidence variant classification thresholds for performance assessment. (**a**) Construction of CardioBoost: (1) After defining gold standard data, (2) the data set was split with a 2:1 proportion into training and test tests. The training set was used for two rounds of cross-validation (CV): first to optimize individually a number of possible machine learning algorithms, and second to select the best-performing tool. (3) AdaBoost was the best-performing algorithm, and forms the basis of CardioBoost. (4) CardioBoost was benchmarked against existing best-in-class tools using the holdout test data, (5) a number of additional independent test sets, and (6) approaches based on association with clinical characteristics of heterozygotes that do not rely on a gold standard classification. (**b**) Illustrative distributions of predicted pathogenicity scores for a set of pathogenic and benign variants obtained by a hypothetical binary classifier. In a clinical context (based on American College of Medical Genetics and Genomics/Association for Molecular Pathology [ACMG/AMP] guidelines), variants are classified into the following categories according to the probability of pathogenicity: disease-causing (probability of pathogenicity [Pr] ≥0.9), benign/likely benign (Pr ≤ 0.1) and a clinically indeterminate group of variants of uncertain significance with low interpretative confidence (0.1 < Pr < 0.9). (**c**) The corresponding confusion matrix with the defined double classification thresholds Pr ≥ 0.9 and Pr ≤ 0.1.

For each variant, we collected 76 functional annotations (Table S6 and Supplementary Methods) as features in our disease-specific variant classification tool. We selected nine classification algorithms including best-in-class representatives of all of the major families of machine learning algorithms, and applied a nested cross-validation^13^ to select the optimal algorithm for our tool. In the inner fivefold cross-validation loop, a candidate classification algorithm was trained in order to optimize its hyperparameters. In the outer tenfold cross-validation loop, the optimized candidate algorithms were compared and the best-performing one was selected (Fig. 1 and Supplementary Methods).

For both conditions, AdaBoost^11^ was selected with the best cross-validated out-of-sample performance (see Supplementary Methods and Tables S7, S8). AdaBoost is a boosting tree classification algorithm combining many decision trees. Each decision tree is learned sequentially to assign more weight to samples misclassified by the previous decision tree, and weighted by its accuracy. Having selected AdaBoost as the basis for our classifier, a predictive model was constructed by training AdaBoost on the whole set of training variants for each disease, named CardioBoost.

### Ethics statement

Training and test data used in the development of the tool were either already in the public domain, or do not constitute personal data, or were obtained with patient consent and/or approval by the following relevant research ethics committees or institutional review boards: South Central–Hampshire B Research Ethics Committee (09/H0504/104), Hammersmith & Queen Charlotte’s Research Ethics Committee (09/H0707/69), Aswan Heart Centre Research Ethics Committee (20160401MYFAHC_HVOL), and SingHealth Centralised Institutional Review Board C (2019/2241).

## RESULTS

### CardioBoost outperforms state-of-the-art genome-wide prediction tools based on overall classification performance measures

To estimate the classifiers’ performance on VUS, we evaluated their classification performances on the holdout test sets. CardioBoost was compared against state-of-the-art genome-wide variant pathogenicity predictors including M-CAP,^14^ REVEL,^15^ CADD,^5^ Eigen,^16^ and PrimateAI,^17^ reported to have leading performance in pathogenicity prediction of rare missense variants. Classification performance was first summarized using the area under the precision recall curve^18^ (PR-AUC) and the area under the receiver operating characteristic curve (ROC-AUC), without relying on a single predefined classification threshold to discriminate disease-causing and benign variants.

**Fig. 2:**
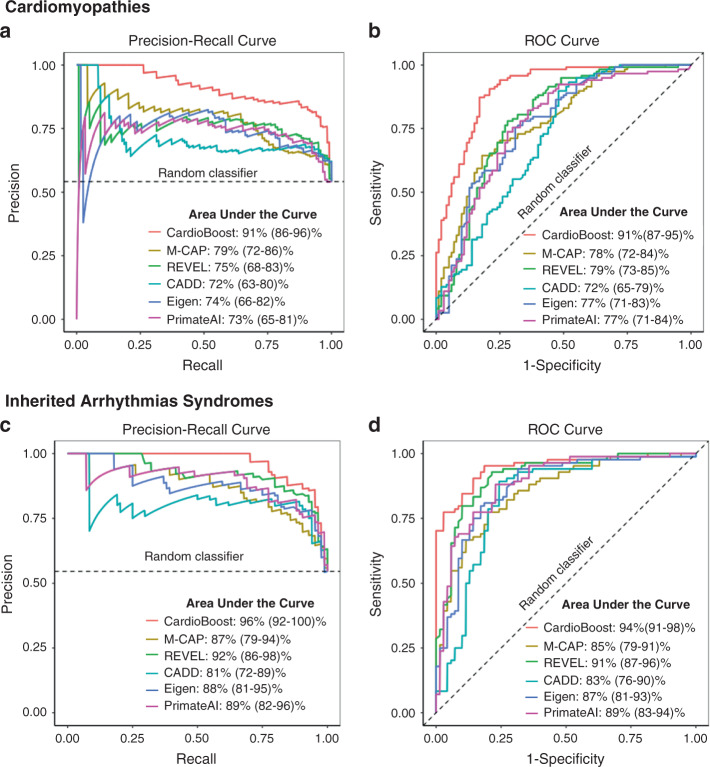
CardioBoost outperforms state-of-the-art genome-wide prediction tools on holdout test data. (**a**, **b**) Precision recall curves and receiver operating characteristic (ROC) curves for cardiomyopathy variant pathogenicity prediction. (**c**, **d**) Precision recall curves and ROC curves for inherited arrhythmia variant pathogenicity prediction. The dashed lines demonstrate the performance of a random classifier.

In both inherited cardiac conditions, CardioBoost achieved the best values in both PR-AUC and ROC-AUC (Fig. 2). The difference in performance was statistically significant for cardiomyopathies, with significantly increased PR-AUC (maximum *P* value = 0.005 between the pairwise statistical comparisons using permutation test) and ROC-AUC (maximum *P* value = 5×10^−6^ between the statistical comparisons using Delong test^19^). Among probabilistic predictors (CardioBoost, M-CAP, REVEL, and PrimateAI), CardioBoost has significantly increased Brier score for both cardiomyopathies (maximum *P* value = 0.005 between the pairwise comparisons via permutation test) and arrhythmia syndromes (maximum *P* value = 0.02 between the pairwise comparisons via permutation test) (Table S9).

In the subsequent benchmarking studies, we specifically demonstrate CardioBoost performances compared with M-CAP and REVEL since they are explicitly trained to distinguish rare disease-causing variants from rare benign ones using ensemble learning approaches comparable with CardioBoost, and their overall classification performances are representative of these state-of-the-art tools shown in the above analysis. As the pathogenicity scores of M-CAP and REVEL were used as input features for CardioBoost, CardioBoost might indirectly expose to variants used in their previous training. This might worsen classification performance if the variants were erroneously classified during upstream training, or lead to inflated performance estimates through overfitting, so we investigated the extent to which these potential limitations influenced CardioBoost performance. CardioBoost was shown to consistently improve on cardiomyopathy- and arrhythmia-specific prediction over existing genome-wide tools both on indirectly “seen” (variants used to train upstream genome-wide learners) and “unseen” (completely novel) data. The overall accuracy of CardioBoost between the unseen and seen data sets is not significantly different for either CM or IAS. (Tables S10, S11 and Supplementary Methods).

### CardioBoost outperforms existing genome-wide prediction tools on high-confidence classification measures

In addition to estimating conventional classification performance, we evaluated performance at thresholds corresponding to accepted levels of certainty required for clinical decision making^1^ (90%; see definitions on Fig. 1b, c and Supplementary Methods). Using these thresholds (disease-causing: probability of pathogenicity (Pr) ≥0.9; benign/likely benign: Pr ≤ 0.1; indeterminate: 0.1 < Pr < 0.9), CardioBoost again outperforms existing genome-wide machine learning variant classification tools when assessed using holdout test data (Table 1).

**Table 1:** CardioBoost outperforms existing genome-wide tools for the classification of holdout test variants.

%	Cardiomyopathies			Arrhythmias		
	CardioBoost	M-CAP	REVEL	CardioBoost	M-CAP	REVEL
Overall accuracy	**63.3**^**a**^	28.4	17.4	**81.2**^**a**^	30.5	37
Proportion of variants classified with high confidence	**70.2**^**a**^	33.9	22	**88.3**^**a**^	33.8	40.3
Accuracy of high-confidence classifications	**90.2**	83.8	79.2	**91.9**	90.4	**91.9**
Proportion of variants with indeterminate classification	**29.8**^**a**^	66.1	78	**11.7**^**a**^	66.2	59.7
TPR	**69.5**^**a**^	41.5	28	**83.3**^**a**^	48.8	65.5
PPV	**86.3**	81.7	76.7	90.9	91.1	**91.7**
TNR	**56**^**a**^	13	5	**78.6**^**a**^	8.6	2.9
NPV	96.6	92.9	**100**	93.2	85.7	**100**

The performance of each tool is reported using the clinically relevant variant classification thresholds: high-confidence disease-causing (Pr ≥ 0.9), high-confidence benign (Pr ≤ 0.1), and indeterminate. For each predictive performance measure (see Supplementary Methods for details) the best algorithm is highlighted in bold. Permutation tests were performed to evaluate whether the performance of CardioBoost was significantly different from the best value obtained by M-CAP or REVEL.

*NPV* negative predictive value, *PPV* positive predictive value, *TNR* true negative rate, *TPR* true positive rate.

^a^*P* value ≤ 0.001.

CardioBoost maximizes the identification of both disease-causing and benign variants. In both conditions, CardioBoost had the highest true positive rate (TPR) (CM 69.5%; IAS 83.3%) and true negative rate (TNR) (CM 56%; IAS 78.6%) (Table 1, *P* value < 0.001). In total, CardioBoost correctly classified 63.3% of cardiomyopathy test variants and 81.2% of arrhythmia test variants with 90% or greater confidence level. The proportions of correctly classified variants are significantly higher (*P* value < 0.001) than those obtained with M-CAP (CM 28.4%; IAS 30.5%) and REVEL (CM 17.4%; IAS 37%). In addition, CardioBoost minimizes the number of indeterminate variants. Only 29.8% of cardiomyopathy test variants and 11.7% of arrhythmia test variants achieved indeterminate scores between 0.1 and 0.9, which were significantly fewer (*P* value < 0.001) than those obtained with M-CAP (CM 66.1%; IAS 66.2%) or REVEL (CM 78%; IAS 59.7%) (Table 1).

Overall, using these thresholds CardioBoost assigned high-confidence classifications to 70.2% of cardiomyopathy test variants, among which 90.2% were correct. For arrhythmias, CardioBoost reported 88.3% of test variants with high confidence, with 91.9% prediction accuracy. The reported results are robust to the choice of classification thresholds. While guidelines propose 90% confidence as appropriate thresholds for likely pathogenic or likely benign classifications, some may advocate a higher confidence threshold. When assessed at a 95% certainty classification threshold, CardioBoost continues to consistently outperform genome-wide tools with significantly (*P* value < 0.001) higher accuracies (Table S12).

CardioBoost is not intended to replace a full expert variant assessment in clinical practice, but for comparative purposes it is informative to consider how classification performance changes under different application contexts. Positive predictive value (PPV) and negative predictive value (NPV) are both dependent on the proportion of pathogenic variants in the variant set being tested, and so it is important to consider how our benchmarking translates to real-world application. Here we used the TPR and TNR calculated on our holdout test set to derive estimates of PPV and NPV for CardioBoost applied in different contexts where the true proportion of pathogenic variants might differ. Our estimation provides a lower bound of PPV and NPV under the assumption that pathogenic variants are fully penetrant. In predictive genetic testing, the limitation of false positive prediction is prioritized, necessitating conservative estimates of PPV. Here we estimate reasonably conservative PPVs and corresponding NPVs of CardioBoost applied in two scenarios: in a diagnostic referral series and in samples from a general population. In a diagnostic laboratory cardiomyopathy referral series, where we estimate approximately 60% rare missense variants found in cardiomyopathy-associated genes to be pathogenic, the PPV and NPV of CardioBoost were estimated at 89% and 96% respectively. By contrast, in a general population, where we estimate the proportion of rare pathogenic variants of these ICC genes are ~1%, the PPV and NPV reach 5% and 99.9%. Similarly, we estimated the performance of CardioBoost in an arrhythmia cohort (PPV: 95%; NPV: 87%) and a general population (PPV: 3%; NPV: 99.9%). This suggests that the predictions of disease-causing variants by CardioBoost are calibrated for high confidence only when applied in a diagnostic context, as would be expected. Classifications are appropriate for variants found in patients, with a reasonable prior probability of pathogenicity (details are described in Supplementary Methods).

Finally, as novel pathogenic variants are more likely to be ultrarare (minor allele frequency <0.01%), we also tested CardioBoost performance on a holdout set of only ultrarare variants and confirmed that it consistently outperforms existing genome-wide tools (Table S13). Its performance on ultrarare variants is comparable with that on rare variants.

### Replication on additional independent test data confirms that CardioBoost improves prediction of disease-causing and benign variants

We collected four additional sets of independent test data to further assess the CardioBoost performance, using variants reported as pathogenic in ClinVar and HGMD^20^ (both databases of aggregated classified variants), a diagnostic laboratory referral series from the Oxford Molecular Genetics Laboratory (OMGL), and a large registry of HCM patients, SHaRe.^21^ When using ClinVar variants to test CM, only variants with two-star review status (i.e., criteria provided, multiple submitters, no conflicts) are included. CardioBoost consistently achieved the highest TPRs: predicting the most disease-causing variants with over 90% certainty (Table 2). On a set of rare variants found in the gnomAD reference data set, which is not enriched for inherited cardiac conditions and hence where the prevalence of disease should be equivalent to the general population, CardioBoost consistently predicts the most variants as benign (Table 2). We also assessed the accuracy of CardioBoost using cell-based functional mapping of amino acid substitutions in calmodulin genes (*CALM1*, *CALM2*, and *CALM3*) from a previous deep mutational scanning (DMS) study.^22^ Averaged over three calmodulin genes, CardioBoost has the significantly highest accuracy to predict the DMS classification (Table 2). CardioBoost also performed the best when assessed at a higher 95% certainty classification threshold (Table S14) and on sets of ultrarare variants (Table S15).

**Table 2:** Evaluation of performance on additional test sets.

	Cardiomyopathies			
	Pathogenic test variants (TPR)			Benign/population test variants (TNR)
	SHaRe (*N* = 129)	ClinVar (*N* = 15)	HGMD (*N* = 145)	gnomAD (*N* = 2003)
CardioBoost	**62.0**^**a**^	**66.7**	**41.4**^**a**^	**51.5**^**a**^
M-CAP	37.2	40.0	22.1	20.3
REVEL	24.0	53.3	22.8	5.6

	Arrhythmias			
	Pathogenic test variants (TPR)		Benign/population test variants (TNR)	Deep mutational scanning (accuracy)
	OMGL (*N* = 77)	HGMD (*N* = 138)	gnomAD (*N* = 1237)	Calmodulin (*N* = 576)
CardioBoost	**88.3**^**a**^	**72.5**^**a**^	**64.3**^**a**^	**29.0**^**a**^
M-CAP	59.7	39.9	9.8	0.3
REVEL	68.8	52.9	2.8	4.2

CardioBoost performance was evaluated against additional variant sets. Four resources provided known pathogenic variants (SHaRe cardiomyopathy registry, ClinVar (two-star submissions), a UK regional genetic laboratory (Oxford Medical Genetics Laboratory [OMGL]) and the Human Gene Mutation Database [HGMD]). Variants found in gnomAD population controls were expected to be predominantly benign. Since gnomAD includes variants seen in the previous ExAC data set that was partially used to train M-CAP and REVEL, we tested against the subset of variants in gnomAD that were not in ExAC. The number of single-nucleotide variants in each set is shown in parentheses. The TPR is reported for pathogenic variant test sets (with threshold Pr ≥ 0.9), and the TNR for benign variant test sets (with threshold Pr ≤ 0.1). We also evaluated the classification accuracies on functional mapping of amino acid substitutions in calmodulin genes obtained through a previous deep functional scanning study. For each performance measure the best algorithm is highlighted in bold. Permutation tests were carried out to evaluate whether the performance of CardioBoost was significantly different from the best value obtained by M-CAP or REVEL.

*TNR *true negative rate, *TPR* true positive rate.

^a^*P* value ≤ 0.001.

### CardioBoost discriminates variants that are highly disease-associated

Since benchmarking against a gold standard variant set may be susceptible to classification errors in the data, we employed two additional approaches to evaluate CardioBoost predictions directly against patient characteristics, to confirm biological and clinical relevance.

**Fig. 3:**
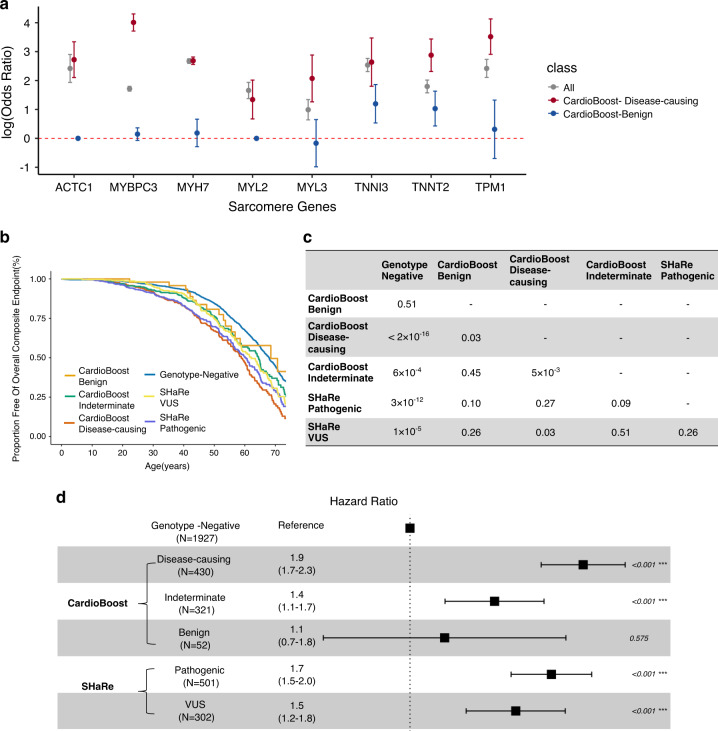
CardioBoost improves prioritization of variants associated with disease and clinical outcomes in patients with hypertrophic cardiomyopathy (HCM). (**a**) We compared the odds ratios (ORs) (on log scale) for three groups of variants: (i) all rare variants, (ii) rare variants predicted disease-causing by CardioBoost (Pr ≥0.9, and excluding those seen in our training data), and (iii) rare variants predicted as benign by CardioBoost (Pr ≤ 0.1, and excluding those seen in our training data). For most of the sarcomere-encoding genes, variants classified as disease-causing by CardioBoost are enriched for disease association, and those classified as benign are depleted, compared with unstratified rare missense variants. (**b**–**d**) CardioBoost variant classification stratifies key clinical outcomes in patients with HCM. Clinical outcomes provide an opportunity to assess classifier performance independent of the labels used in the gold standard training data. (**b**) Kaplan–Meier event-free survival curves are shown for patients in the SHaRe cardiomyopathy registry, stratified by genotype as interpreted by CardioBoost. The patients carrying variants seen in the CardioBoost training set were excluded from this analysis. Patients with predicted disease-causing variants in sarcomere-encoding genes have more adverse clinical events compared with patients without sarcomere-encoding variants (“genotype-negative”), and compared with patients with sarcomere-encoding variants classified as benign. Survival curves stratified by variants as adjudicated by experts (marked in figure with prefix “SHaRe”) are shown for comparison. The composite endpoint comprised the first incidence of any component of the ventricular arrhythmic or heart failure composite endpoint, atrial fibrillation, stroke or death. (**c**) *P* values of the log-rank test in the pairwise comparisons of Kaplan–Meier survival curves. (**d**) Forest plot displays the hazard ratio (with confidence interval) and *P* value of tests comparing patients’ survival stratified by CardioBoost classification and SHaRe experts’ classification based on Cox proportional hazards models.

First, we directly assessed the strength of the association between the specified disease and rare variants stratified by the different tools. We compared the proportions of rare missense variants in a cohort of 6327 genetically characterized patients with HCM, from the SHaRe registry,^21^ with 138,632 reference samples from gnomAD v2.0 (Fig. 3a). We calculated the odds ratio (OR) for all rare variants observed in each sarcomere gene, and for variants stratified by CardioBoost, M-CAP, and REVEL after excluding variants seen in our training data.

For seven of the eight CM-associated genes (*MYH7*, *TNNI3*, *TPM1*, *ACTC1*, *TNNT2*, *MYBPC3*, and *MYL3*), the OR for variants prioritized by CardioBoost (i.e., predicted disease-causing with Pr ≥ 0.9) was greater than the baseline OR (including all observed variants without discriminating disease-causing and benign variants), indicating that the tool is discriminating a set of variants more strongly associated with the disease. For three genes (*TPM1*, *TNNT2*, *MYBPC3*), the difference was statistically significant (*P* value < 0.05). Concordantly, variants in seven of the eight sarcomere genes predicted as benign have significantly decreased association with disease compared with the baseline OR (*P* value < 0.05). By contrast, M-CAP or REVEL did not show any demonstrable difference in disease ORs between predicted disease-causing and predicted benign variants (Table S16).

### CardioBoost variant classification is associated with adverse clinical outcome

As a further assessment independent of gold standard classification, we tested the association of variants stratified by CardioBoost with clinical outcomes in the same cohort of patients. Patients with HCM who carry known pathogenic variants in genes encoding sarcomeric proteins have been shown to follow an adverse clinical course compared with “genotype-negative” individuals (no rare pathogenic variant or VUS in a sarcomere-encoding gene, and no other pathogenic variant identified),^21,23,24^ with a higher burden of adverse events. Patients carrying benign variants in HCM-associated genes would be expected to follow a similar trajectory to those genotype-negative patients.

We evaluated clinical outcomes in a subset of the SHaRe cohort comprising 803 HCM patients each with a rare missense variant in a sarcomere-encoding gene, and 1927 genotype-negative HCM patients, after excluding all patients carrying variants that were seen in the CardioBoost training set. We compared event-free survival (i.e., age until the first occurrence of a composite adverse clinical outcome including heart failure events, arrhythmic events, stroke, and death) of these patients, stratified by CardioBoost-predicted pathogenicity (the full definition of a composite adverse clinical outcome is described in Supplementary Methods).

CardioBoost classification stratifies novel variants with significantly different patient survival curves (Fig. 3b–d). Patients carrying variants predicted as disease-causing (CardioBoost disease-causing) were likely to have earlier onset and a higher adverse event rate than those without identified rare variants (CardioBoost disease-causing vs. genotype-negative: *P* value < 2×10^−16^; hazard ratio [HR] = 1.9), or those with variants predicted to be benign (CardioBoost disease-causing vs. CardioBoost benign: *P* value = 0.03; HR = 1.7). The probability of developing the overall composite outcome by age 60 is 54% (95% CI: 46–59%) for CardioBoost disease-causing patients, versus 33% (95% CI: 30–35%) for genotype-negative patients. By contrast, groups stratified by M-CAP or REVEL variant classification did not show significantly different event-free survival time (M-CAP disease-causing vs. M-CAP benign: *P* value = 0.31; REVEL disease-causing vs. REVEL benign: *P* value = 0.30) (Fig. S1).

## DISCUSSION

Our results show that in silico prediction of variant pathogenicity for inherited cardiac conditions is improved within a disease-specific framework trained using expert-curated interpreted variants. This is demonstrated through improved classification performance, stronger disease association, and significantly improved stratification of patient outcomes over published genome-wide tools.

There are several factors that may contribute to improved performance for a gene- and disease-specific classifier like CardioBoost over genome-wide tools. First, the use of disease-specific labels could decrease the false prediction of benign variants as disease-causing. A variant causative of one Mendelian dominant disorder may be benign with respect to a different disorder (associated with the same gene), if the conditions result from distinct molecular pathways. Since genome-wide tools are trained on universal labels (i.e., whether a variant ever causes any diseases), they would be expected to yield false positive predictions in the context of specific diseases. Second, while the representative genome-wide tools M-CAP and REVEL are trained on variants from HGMD curated from literature, CardioBoost is trained on high-quality expert-curated variants, thus reducing label bias and increasing the prediction performances. Third, as the genome-wide tools are trained across the genome, the learning function that maps the input features into the pathogenicity score is fitted using the training samples from all genes in the genome. However, different genes may have different mapping functions, for example related to different molecular mechanisms. Restricting to a set of well-defined disease-related genes may exclude influences from other unrelated genes.

We might expect a gene disease–specific model would most accurately represent the genotype–phenotype relationship. However, there is a tradeoff between the size of available training data and the specialization of classification tasks. Here, CardioBoost groups together genes for two sets of closely related disorders, including three genes in which variants with different functional consequences lead to distinct phenotypes in our training set (i.e., *SCN5A*, *TNNI3*, *MYH7*). This is a potential limitation, since we hypothesize that distinct functional consequences might optimally be modeled separately. We explored alternative models for cardiomyopathy classifiers, for which our training data set is larger than for arrhythmias. Two disease-specific models (HCM-specific and DCM-specific) and three gene syndrome–specific models (*MYH7*-HCM-specific, *MYH7*-DCM-specific, and *MYBPC3*-HCM-specific) with the largest training data size were built and compared (Table S17). None of the alternative models had comparable performance with the combined cardiomyopathy model. We therefore conclude that given the current availability of training data, a cardiomyopathy-specific classifier provides the best empirical balance between grouping variants with similar phenotypic effects and making use of relatively large training data set. It improves prediction both over genome-wide models that entirely ignore variants’ phenotypic effects, and over gene disease–specific models for which there are insufficient training data. We therefore adopted the broadly disease-specific models as our final classifier, but anticipate that complete separation of distinct phenotypes may be advantageous when more training data become available in the future.

CardioBoost natively outputs a continuous probability of pathogenicity that is directly interpretable. Users may therefore define their own confidence thresholds according to intended application. The posterior probability can also be updated by incorporating further evidence, such as linkage scores calculated from the evaluation of segregation in a family.

There are several potential limitations and avenues for future refinement. First, we have only considered the prediction of pathogenicity for missense variants thus far. The inclusion of different classes of variants in disease-specific model is challenging since there are limited high-confidence training data for nonmissense variants.

A second key limitation of CardioBoost is that it does not consider all relevant lines of evidence, and therefore it is not intended to serve as a tool for comprehensive assessment of variant pathogenicity. Some evidence types are limited by availability such as population allele frequency data and segregation data. Others could not be systematically included into a machine learning framework either because they are not well structured as in the case of functional data, de novo data, and allelic data, or they are too sparse. For example, many variants lack experimental data, and the precise population allele frequency of many variants is unknown, though this implies significant rarity. In our training data, 45% of variants in cardiomyopathies and 44% of variants in arrhythmias were not seen in the gnomAD control population. Here, we do not include allele frequencies in gnomAD as a predictive feature since the relation between variant pathogenicity and allele frequency scale beyond current observation is clearly unknown.

For these reasons, while we show advantages of the proposed model for variant classification in known disease genes over existing genome-wide tools, we emphasize that CardioBoost is not intended for use as a standalone clinical decision tool, or as a replacement for the existing ACMG/AMP guidelines for variant interpretation. Rather, in its current form it could provide a numerical value for evidence PP3 (“Multiple lines of computational evidence support a deleterious effect on the gene/gene product”) and BP4 (“Multiple lines of computational evidence suggest no impact on gene/gene product”) that is more reliable and accurate than existing genome-wide variant classifiers in the context of inherited cardiac conditions. We suggest that CardioBoost high-confidence classifications might appropriately activate PP3 (Pr > 0.9) and BP4 (Pr < 0.1). It is interpreted as the supporting evidence being activated with at least 90% confidence.

The widely adopted ACMG/AMP framework is semiquantitative, but one limitation is that the weightings applied to different rules are not all evidence-based or proven to be mathematically well calibrated. We do anticipate that, with more training data and robust validation, quantitative tools like CardioBoost will prove informative for variant interpretation, and will carry more weight in a quantitative decision framework than the current ACMG/AMP PP3 and BP4 rule affords.

While CardioBoost improves on existing tools, there remain a substantial number of variants receiving indeterminate classification by CardioBoost at high-confidence classification thresholds (Table 1: CM 29.8% IAS 11.7%). We anticipate that additional relevant functional annotations and accumulation of further gold standard interpreted data will continue to improve in silico prediction over time.

While CardioBoost performs well overall, the prediction performance and confidence vary for different genes according to the size of the training/test set for that gene. Five genes account for the majority of genetically explained cardiomyopathy and long QT (*MYH7*, *MYBPC3*, *KCNQ1*, *KCNH2*, *SCN5A*), resulting in narrower prediction confidence intervals. For other genes, the gold standard data remain relatively sparse (Fig. S2), resulting in wider prediction confidence intervals. Classifications of variants in these genes should be considered with appropriate care.

In conclusion, as exemplified in inherited cardiac conditions, we have substantiated that a disease-specific variant classifier improves the in silico prediction of variant pathogenicity over the best-performing genome-wide tools. Our study also emphasizes the pitfalls of relying on genome-wide variant classifiers and the necessity to develop disease-specific variant classifiers to accurately interpret variant pathogenicity on specific phenotypes and diseases. We also highlight the need to evaluate variant pathogenicity prediction in clinical settings including accuracies on high-confidence classification thresholds equivalent to accepted certainty required for clinical decision making, variants’ association with disease, and patients’ clinical outcomes. To support accurate variant interpretation in inherited cardiac conditions, we provide precomputed pathogenicity scores for all possible rare missense variants in genes associated with inherited cardiomyopathies and arrhythmias (https://www.cardiodb.org/cardioboost/). The demonstrated development and evaluation framework could be applicable to develop accurate disease-specific variant classifiers and improve variant interpretation in a wide range of Mendelian disorders.

## Supplementary information

Supplementary Material

## Data Availability

The source code and data to reproduce our model development and validation analyses can be found on GitHub at https://github.com/ImperialCardioGenetics/CardioBoost_manuscript.
