# Supplementary material for: Disease-specific variant pathogenicity prediction significantly improves variant interpretation in inherited cardiac conditions
Source: Genet Med. 2020 Oct 13;23(1):69–79. doi: 10.1038/s41436-020-00972-3 (PMC7790749; doi:10.1038/s41436-020-00972-3)
Supplement: Supplementary file 1 — Supplementary Material [file 41436_2020_972_MOESM1_ESM.pdf]

Supplementary Figures

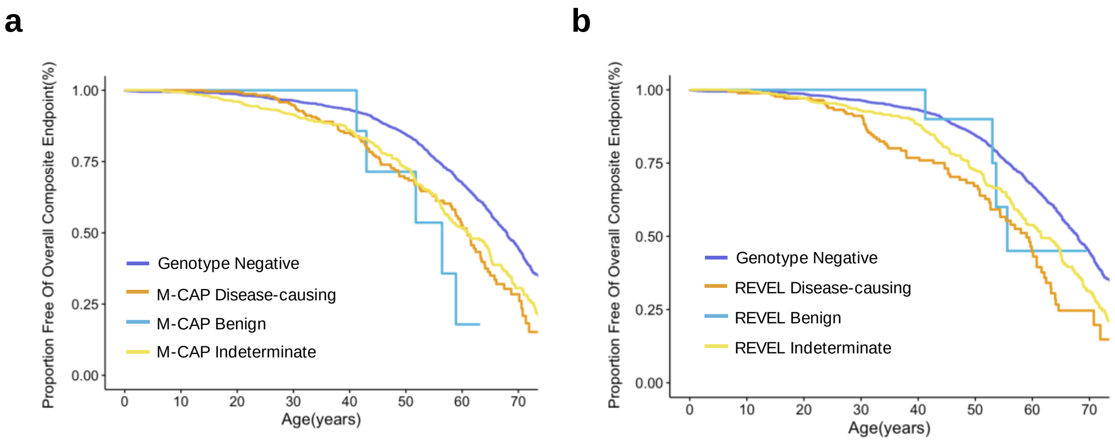

Figure S1. Variants classification by state-of-the-art genome-wide tools M-CAP and REVEL did not show to stratify the survival outcomes of patients. (**a**) Kaplan-Meier event-free survival curves for patients in the SHaRe cardiomyopathy registry, stratified by genotype as interpreted by M-CAP. The patients with variants predicted disease-causing by M-CAP did not have significantly different survival time compared to those with predicted benign variants (log-rank test *P*-value = 0.31). (**b**) Kaplan-Meier event-free survival curves for patients in the SHaRe cardiomyopathy registry, stratified by genotype as interpreted by REVEL. Patients with predicted disease-causing variants by REVEL did not have significantly different survival time compared to those with predicted benign variants (log-rank test *P*-value = 0.30).

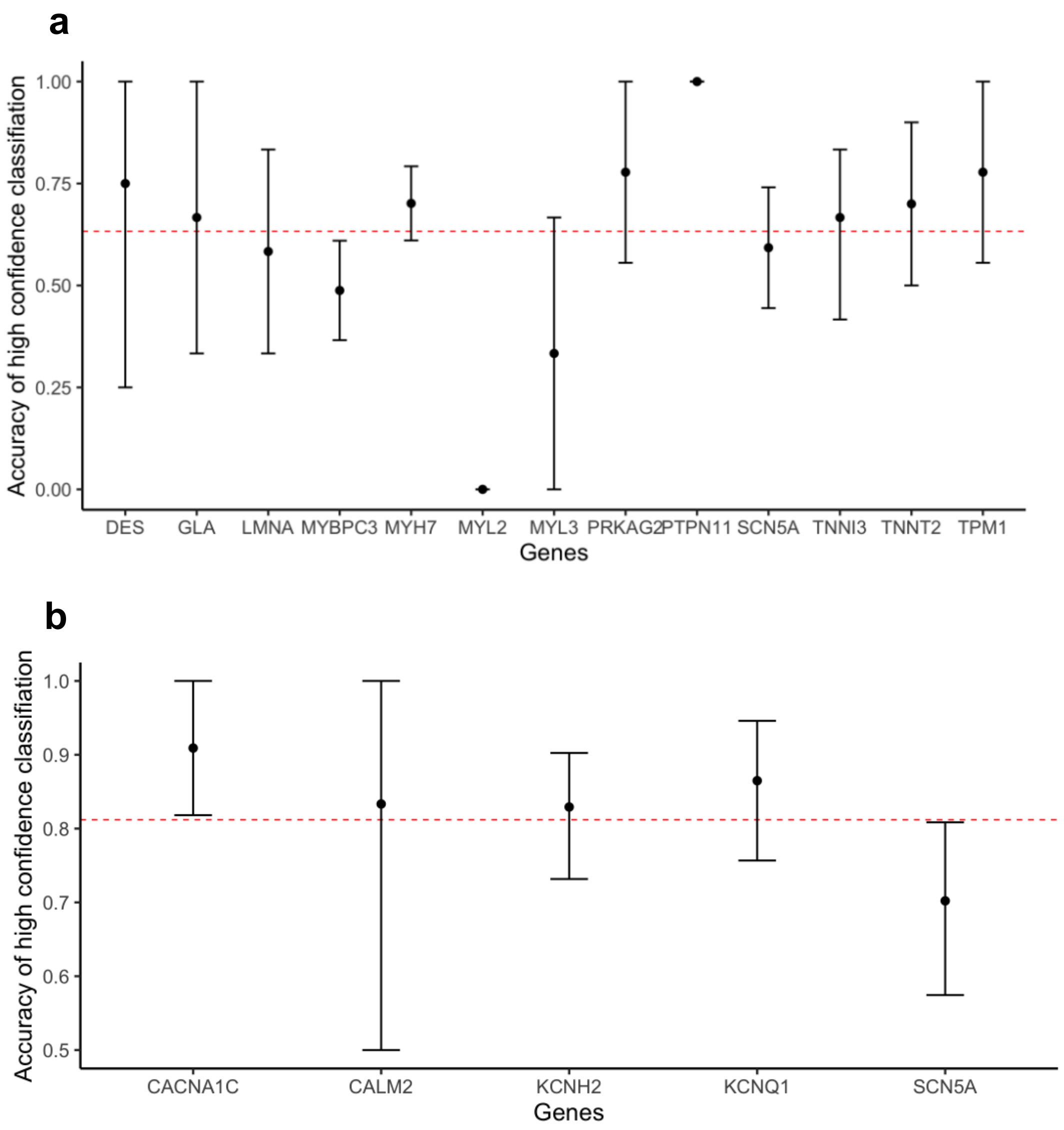

Figure S2. Variant classification performance per gene. The accuracy of high-confidence classification and its 90% bootstrap CI (n=1,000 times) are calculated per gene for (a) cardiomyopathies and (b) arrhythmias. The red dashed lines indicate the overall accuracies of variant classification at disease-level (extracted from Table 1). To be noticed, here the bootstrap CI is subjected to the size of test variants for each gene. Only genes with more than one test variants are considered in the analysis. Particular care should be taken for genes with wider confidence interval in using CardioBoost for variant classification.

List of Supplementary Tables

Supplementary Table 1. Cardiomyopathy-associated genes included in the study.

Supplementary Table 2. Arrhythmia-associated genes included in the study.

Supplementary Table 3. Data sets used for the development of CardioBoost.

Supplementary Table 4. The training data and hold-out test data grouped by gene used by CardioBoost for cardiomyopathies.

Supplementary Table 5. The training data and hold-out test data grouped by gene used by CardioBoost for arrhythmias.

Supplementary Table 6. Input variant features collected from existing computational tools.

Supplementary Table 7. Cross-validated out-of-sample performance for cardiomyopathy variant pathogenicity prediction.

Supplementary Table 8. Cross-validated out-of-sample performances for arrhythmia variant pathogenicity prediction.

Supplementary Table 9. Brier Scores to compare performances of probabilistic variant pathogenicity predictions in the hold-out test data set.

Supplementary Table 10. Performance comparison on variants “unseen” and indirectly “seen” in the hold-out test data set for cardiomyopathy variant pathogenicity prediction.

Supplementary Table 11. Performance comparison on variants “unseen” and indirectly “seen” in the hold-out test data set for arrhythmia variant pathogenicity prediction.

Supplementary Table 12. CardioBoost outperforms existing genome-wide tools for the classification of hold-out test variants using 95%-certainty thresholds.

Supplementary Table 13. Comparison of classification performances on the hold-out test data set with minor allele frequency < 0.01%.

Supplementary Table 14. Evaluation of performances on additional test sets using 95%-certainty threshold.

Supplementary Table 15. Evaluation of performances on additional test sets with minor allele

frequency < 0.01%.

Supplementary Table 16. CardioBoost variant classification stratifies variants with increased disease Odds Ratio for sarcomere-encoding genes.

Supplementary Table 17. Comparison of out-of-sample classification performances for alternative disease-specific classification models.

Supplementary Table 1. Cardiomyopathy-associated genes included in the study.

Gene symbol	Phenotype	Ensemble gene ID	Ensemble transcript ID	Ensemble protein ID
ACTC1	HCM[1]	ENSG00000159251	ENST00000290378	ENSP00000290378
DES	DCM[3] (syndromic)	ENSG00000175084	ENST00000373960	ENSP00000363071
GLA	HCM[3] (syndromic)	ENSG00000102393	ENST00000218516	ENSP00000218516
LAMP2	HCM[3] (syndromic)	ENSG00000005893	ENST00000200639	ENSP00000200639
LMNA	DCM	ENSG00000160789	ENST00000368300	ENSP00000357283
MYBPC3	HCM	ENSG00000134571	ENST00000545968	ENSP00000442795
MYH7	HCM & DCM[1]	ENSG00000092054	ENST00000355349	ENSP00000347507
MYL2	HCM	ENSG00000111245	ENST00000228841	ENSP00000228841
MYL3	HCM	ENSG00000160808	ENST00000395869	ENSP00000379210
PLN	Intrinsic CM[2]	ENSG00000198523	ENST00000357525	ENSP00000350132
PRKAG2	HCM[3] (syndromic)	ENSG00000106617	ENST00000287878	ENSP00000287878
PTPN11	HCM[3] (syndromic)	ENSG00000179295	ENST00000351677	ENSP00000340944
SCN5A	DCM	ENSG00000183873	ENST00000333535	ENSP00000328968

TNNI3	HCM & DCM[1]	ENSG00000129991	ENST00000344887	ENSP00000341838
TNNT2	HCM[1]	ENSG00000118194	ENST00000367318	ENSP00000356287
TPM1	HCM[1]	ENSG00000140416	ENST00000403994	ENSP00000385107

[1] While there are several genes in this table that have been associated with more than one type of cardiomyopathy, e.g. with different variants causing HCM and DCM, our training and test data included variants associated with just one type of cardiomyopathy for all genes except *MYH7 and TNNI3.* For *MYH7* and *TNNI3*, the output of CardioBoost should be interpreted as "probability of pathogenicity for HCM or DCM". For other genes associated with more than one subtype the classifier is trained for a particular disease only, and should be interpreted as such.

[2] The cardiomyopathic phenotype associated with variants in *PLN* does not fit neatly into the clinical definitions of HCM and DCM, so it has been classified under the broader umbrella of intrinsic cardiomyopathy[1].

[3] These conditions typically present with cardiomyopathy in the context of a broader syndromic phenotype, but may also present with isolated heart disease[1].

Supplementary Table 2. Arrhythmia-associated genes included in the study.

Gene symbol	Phenotype	Ensemble gene ID	Ensemble transcript ID	Ensemble protein ID
CACNA1C	Timothy Syndrome (LQT)	ENSG00000151067	ENST00000399655	ENSP00000382563
CALM1	LQT	ENSG00000198668	ENST00000356978	ENSP00000349467
CALM2	LQT	ENSG00000143933	ENST00000272298	ENSP00000272298
CALM3	LQT	ENSG00000160014	ENST00000291295	ENSP00000291295
KCNH2	LQT	ENSG00000055118	ENST00000262186	ENSP00000262186
KCNQ1	LQT	ENSG00000053918	ENST00000155840	ENSP00000155840
SCN5A	LQT & BrS[1]	ENSG00000183873	ENST00000333535	ENSP00000328968

(LQT = Long QT syndrome; BrS = Brugada syndrome)

[1]For *SCN5A*, the output of CardioBoost should be interpreted as “probability of pathogenicity for LQT or BrS”.

Supplementary Table 3. Data sets used for the development of CardioBoost. The number of missense variants in the training and hold-out test datasets is shown for two groups of inherited cardiac conditions.

	Cardiomyopathies			Arrhythmias		
	Pathogenic	Benign	Total	Pathogenic	Benign	Total
Training data set	238	202	440	168	158	326
Test data set	118	100	218	84	79	163
**Total**	**356**	**302**	**658**	**252**	**237**	**489**

Supplementary Table 4. The training data and hold-out test data grouped by gene used by CardioBoost for cardiomyopathies. The number of missense variants in the training and hold-out test datasets is shown for each gene.

Gene symbol	Training		Test	
	Benign	Pathogenic	Benign	Pathogenic
ACTC1	0	2	1	0
DES	13	3	4	0
GLA	5	5	3	3
LAMP2	5	2	1	0
LMNA	6	10	5	7
MYBPC3	47	19	27	14
MYH7	25	125	13	64
MYL2	1	11	1	1
MYL3	4	3	2	1
PLN	1	2	1	0
PRKAG2	14	2	7	2
PTPN11	8	1	2	1
SCN5A	55	2	27	0
TNNI3	6	23	4	8

TNNT2	8	14	2	8
TPM1	4	14	0	9
**Total**	**202**	**238**	**100**	**118**

Supplementary Table 5. The training data and hold-out test data grouped by gene used by CardioBoost for arrhythmias. The number of missense variants in the training and hold-out test datasets is shown for each gene.

Gene symbol	Training		Test	
	Benign	Pathogenic	Benign	Pathogenic
CACNA1C	37	4	19	3
CALM1	0	5	0	1
CALM2	0	4	0	6
CALM3	0	3	0	0
KCNH2	33	54	19	22
KCNQ1	12	55	6	31
SCN5A	58	43	26	21
**Total**	**140**	**168**	**70**	**84**

Supplementary Table 6. Input variant features collected from existing computational tools.

Features	Data type	Description
Grantham score	Integer	Substitution matrix scoring the distance from one amino acid to the other
BLOSUM62	Integer	
PAM250	Integer	
SIFT	Float	Estimate intolerance to variation from closely-related species sequence alignment
Polyphen2	Float x 2	Machine learning method to predict functional effects using structural and sequence features
LRT_score	Float	The original LRT two-sided *P*-value
MutationTaster	Float	Bayes classifier used to predict pathogenicity of variants
MutationAssessor	Float	Predicts functional impact of amino acid substitutions
FATHMM	Float	HMM model to predict functional effects of variants
PROVEAN	Float	Predicts whether an amino acid substitution or indel has an impact on the biological function of a protein
VEST3	Float	Machine learning method to predict variant functional effects
CADD	Float	SVM models to predict pathogenicity for coding and non-coding variants

DANN	Float	Scores whole-genome variants by training a deep neural network
FATHMM-MKL	Float	Machine learning method to predict variant functional effects
MetaSVM	Float	Machine learning method to predict SNVs functional effects
MetaLR	Float	Very similar to MetaSVM, but better interpretable
Eigen	Float x 2	Unsupervised machine learning methods to predict function effects of coding and non-coding variants
M-CAP	Float	Gradient boosting tree to predict functional effects of missense variants
REVEL	Float	Random Forest to predict functional effects of missense variants
GERP++	Float	Identify constrained elements in multiple alignments
PhyloP	Float x 2	Base pair level multi species conservation
Integrated_fitcons	Float	Estimate of fitness consequences
PhastCons	Float x 2	Regional multi species conservation metric
SiPhy	Float	Detect bases under selection based on multiple alignments
paraZscore	Float	Estimate conservation across related proteins within-species from gene paralog

paraZscore_exist	Integer	Indicate whether the paraZscore of a missense variant is available
misbadness	Float	Measures the increased deleteriousness of amino acid substitutions when they occur in missense-constrained regions
misbadness_exist	Integer	Indicate whether the misbadness score of a variant is available
MPC	Float	Integrated score of misbadness, polyphen-2 and constraint

Supplementary Table 7. Cross-validated out-of-sample performance for cardiomyopathy variant pathogenicity prediction. We compared nine classification algorithms including best-in-class representatives of all of the major families of machine learning algorithms. AdaBoost was selected with the best cross-validated out-of-sample performance. PR-AUC: Area under the Precision Recall Curve; ROC-AUC: Area under the Receiver Operating Curve; MCC: Mathew Correlation Coefficient.

Method category	Algorithm	PR-AUC (%)	ROC-AUC (%)	Brier score	MCC
Regression	GLMNET	90	88	0.15	0.10
Tree-based	CART	83	81	0.18	0.43
	RF	90	89	0.14	0.36
	BART	91	89	0.14	0.38
Boosting-based	XGBoost	90	87	0.15	0.51
	GBM	87	87	0.15	0.43
	**Adaboost**	**90**	**88**	**0.14**	**0.58**
Other classification algorithms	KNN	89	88	0.15	0.43
	SVM-RBF	89	87	0.14	0.36
Existing genome-wide classification tools	M-CAP	80	79	0.19	0.35
	REVEL	79	81	0.19	0.25

Supplementary Table 8. Cross-validated out-of-sample performances for arrhythmia variant pathogenicity prediction. We compared nine classification algorithms including best-in-class representatives of all of the major families of machine learning algorithms. AdaBoost was selected with the best cross-validated out-of-sample performance.

Method category	Algorithm	PR-AUC (%)	ROC-AUC (%)	Brier score	MCC
Regression	GLMNET	91	91	0.12	0.22
Tree-based	CART	82	86	0.14	0.56
	RF	93	92	0.10	0.45
	BART	93	92	0.11	0.43
Boosting-based	XGBoost	88	90	0.12	0.56
	GBM	87	89	0.12	0.60
	**Adaboost**	**90**	**90**	**0.13**	**0.65**
Other classification algorithm	KNN	92	91	0.12	0.45
	SVM-RBF	92	92	0.10	0.47
Existing genome-wide classification tools	M-CAP	81	85	0.16	0.38
	REVEL	89	90	0.17	0.59

Supplementary Table 9. Brier Scores to compare performances of probabilistic variant pathogenicity predictions in the hold-out test data set.

	Cardiomyopathies	Arrhythmias
CardioBoost	**0.12**	**0.09**
M-CAP	0.20	0.17
REVEL	0.19	0.17
PrimateAI	0.21	0.18

Supplementary Table 10. Performance comparison on variants "unseen" and indirectly "seen" in the hold-out test data set for cardiomyopathy variant pathogenicity prediction. To assess whether bias is introduced in evaluating variants previously used in the training of M-CAP and REVEL, the performance of CardioBoost on wholly "unseen" data (not used in the training of M-CAP and REVEL), and indirectly "seen" data" (used in the training of M-CAP and REVEL) were compared with M-CAP and REVEL. For each predictive performance measure (see **Supplementary Methods** for details) the best algorithm is highlighted in bold.

	"Unseen" data $N_{pathogenic}$ = 41 N_{benign} = 24			"Seen" data $N_{pathogenic}$ = 77 N_{benign} = 76		
	CardioBoost (%)	M-CAP (%)	REVEL (%)	CardioBoost (%)	M-CAP (%)	REVEL (%)
PR-AUC	**90.2**	80.2	73.8	**91.8**	78.6	76.7
ROC-AUC	**86.3**	71.1	70.2	**92.1**	79.8	81.9
Brier Score	**13.4**	21.5	19.5	**11.8**	19.0	19.2
Overall Accuracy	**60.0**	30.8	12.3	**64.7**	27.5	19.6
Proportion of variants classified with high confidence	**69.2**	40.0	20.0	**70.6**	31.4	22.9

Accuracy of high-confidence classifications	**86.7**	76.9	61.5	**91.7**	87.5	85.7
Proportion of variants with indeterminate classifications	**30.8**	60.0	80.0	**29.4**	68.6	77.1
TPR	**70.7**	43.9	19.5	**68.8**	40.3	32.5
PPV	**82.9**	75.0	61.5	**88.3**	86.1	83.3
TNR	**41.7**	8.3	0.0	**60.5**	14.5	6.6
NPV	**100.0**	**100.0**	NA[1]	95.8	91.7	**100.0**

1 No variants are classified as benign by REVEL.

Supplementary Table 11. Performance comparison on variants "unseen" and indirectly "seen" in the hold-out test data set for arrhythmia variant pathogenicity prediction. To assess whether bias is introduced in evaluating variants previously used in the training of M-CAP and REVEL, the performance of CardioBoost on entirely "unseen" data (not used in the training of M-CAP and REVEL), and indirectly "seen" data" (used in the training of M-CAP and REVEL) were compared with M-CAP and REVEL. For each predictive performance measure (see **Supplementary Methods**for details) the best algorithm is highlighted in bold.

	"Unseen" data $N_{pathogenic}$ = 17 N_{benign} = 18			"Seen" data $N_{pathogenic}$ = 67 N_{benign} = 52		
	CardioBoost (%)	M-CAP (%)	REVEL (%)	CardioBoost (%)	M-CAP (%)	REVEL (%)
PR-AUC	**94.4**	82.2	87.1	**96.8**	88.6	93.1
ROC-AUC	**94.1**	85.6	86.3	**95.0**	84.6	92.6
Brier Score	**12.2**	15.9	20.6	**9.3**	17.4	16.2
Overall Accuracy	**80.0**	34.3	28.6	**81.5**	29.4	39.5
Proportion of variants classified with high confidence	**88.6**	40.0	34.3	**88.2**	31.9	42.0

Accuracy of high-confidence classifications	**90.3**	85.7	83.3	92.4	92.1	**94.0**
Proportion indeterminate classifications	**11.4**	60.0	65.7	**11.8**	68.1	58.0
TPR	**88.2**	70.6	58.8	**82.1**	43.3	67.2
PPV	**88.2**	85.7	83.3	91.7	93.5	**93.8**
TNR	**72.2**	0.0	0.0	**80.8**	11.5	3.8
NPV	**92.9**	NA	NA	93.3	85.7	**100.0**

Supplementary Table 12. CardioBoost outperforms existing genome-wide classification tools for the classification of hold-out test variants using 95%-certainty thresholds. While 90% is defined as a high-confidence threshold for clinical action in the ACMG/AMP guidelines, some may advocate a more stringent approach. We therefore assessed the performance of each tool using more stringent values for clinically relevant variant classification thresholds: high-confidence disease-causing (Pr ≥ 0.95), high-confidence benign (Pr ≤ 0.05), and indeterminate. For each predictive performance measure (see **Supplementary Methods** for details) the best algorithm is highlighted in bold. Permutation tests were performed to evaluate whether the performance of CardioBoost was significantly different from the best value obtained by M-CAP or REVEL (significance levels: ****P*-value ≤ 0.001, ***P*-value ≤ 0.01, **P*-value ≤ 0.05).

(%)	Cardiomyopathies			Arrhythmias		
	CardioBoost	M-CAP	REVEL	CardioBoost	M-CAP	REVEL
Overall accuracy	**54.6*** **	16.5	7.3	**78.6*** **	7.8	22.1
Proportion of variants classified with high confidence	**60.1*** **	18.8	10.1	**85.1*** **	8.4	23.4
Accuracy of high confidence classifications	**90.8**	87.8	72.7	92.4	92.3	**94.4**
Proportion of variants with	**39.9*** **	81.2	89.9	**14.9*** **	91.6	76.6

indeterminate classification						
TPR	**62.7*** **	24.6	11.9	**79.8*** **	11.9	39.3
PPV	**87.1**	85.3	70.0	91.8	90.9	**93.9**
TNR	**45.0*** **	7.0	2.0	**77.1*** **	2.9	1.4
NPV	97.8	**100.0**	**100.0**	93.1	**100.0**	**100.0**

Supplementary Table 13. Comparison of classification performance on the hold-out test data set with minor allele frequency < 0.01%. As novel pathogenic variants are more likely to be ultra-rare, CardioBoost was tested on the hold-out set of only ultra-rare variants and was confirmed to have comparable performance with that on rare variants. The performance of each tool is reported using the 90% high-confidence variant classification thresholds: high confidence disease-causing (Pr ≥ 0.90), high confidence benign (Pr ≤ 0.10), and indeterminate. For each predictive performance measure (see **Supplementary Methods** for details) the best algorithm is highlighted in bold. Permutation tests were performed to evaluate whether the performance of CardioBoost was significantly different from the best value obtained by M-CAP or REVEL (significance levels: ***P-value ≤ 0.001, **P-value ≤ 0.01, *P-value ≤ 0.05).

	Cardiomyopathies			Arrhythmias		
	CardioBoost (%)	M-CAP (%)	REVEL (%)	CardioBoost (%)	M-CAP (%)	REVEL (%)
Classification performance measures						
PR-AUC	**93***	85	81	**97**	90	95
ROC-AUC	**91*****	79	79	**95**	86	93
Brier Score	**0.11***	0.18	0.17	**0.09**	0.15	0.14
90% high-confidence classification performance measures						
Overall accuracy	**64.9*****	30.9	19.7	**83.6*****	33.6	42.5
Proportion of variants classified with high confidence	**71.3*****	35.6	22.9	**93.3*****	93.8	95

Accuracy of high confidence classifications	**91.0**	86.6	86	93.3	93.8	**95**
Proportion of variants with indeterminate classification	**28.7*** **	64.4	77.1	**6.7*** **	65.2	53.3
TPR	**70.1*** **	41.9	28.2	**85.4*** **	50	67.1
PPV	**89.1**	86	84.6	94.6	**95.3**	94.8
TNR	**56.3*** **	12.7	5.6	**80.8*** **	7.7	3.8
NPV	95.2	90	**100**	91.3	80	**100**

Supplementary Table 14. Evaluation of performances on additional test sets using 95%-certainty threshold.

(%)	Cardiomyopathies			
	Pathogenic test variants (TPR)			Benign test variants (TNR)
	SHaRe (N = 129)	ClinVar (N = 15)	HGMD (N = 145)	gnomAD (N = 2,003)
CardioBoost	**51.2*** **	**60.0***	**33.8*** **	**44.2*** **
M-CAP	19.4	13.3	9.0	9.9
REVEL	6.2	6.7	6.9	2.6

(%)	Arrhythmias			
	Pathogenic test variants (TPR)		Benign test variants (TNR)	Deep Mutational Scanning (Accuracy)
	OMGL (N = 77)	HGMD (N = 138)	gnomAD (N = 1,237)	Calmodulin (N = 576)
CardioBoost	**87.0*** **	**71.0*** **	**61.3*** **	**25.7*** **
M-CAP	23.4	18.8	4.3	0
REVEL	28.6	23.9	1.2	0.3

Supplementary Table 15. Evaluation of performances on additional test sets with minor allele frequency < 0.01%.

(significance levels: ***P-value ≤ 0.001, **P-value ≤ 0.01, *P-value ≤ 0.05).

(%)	Cardiomyopathies			
	Pathogenic test variants (TPR)			Benign test variants (TNR)
	SHaRe (N = 129)	ClinVar (N = 14)	HGMD (N = 143)	gnomAD (N = 1,999)
CardioBoost	**62.0*** **	**71.4***	**42.0*** **	**51.5*** **
M-CAP	37.2	42.9	22.4	20.3
REVEL	24.0	57.1	23.1	5.7

(%)	Arrhythmias			
	Pathogenic test variants (TPR)		Benign test variants (TNR)	Deep Mutational Scanning (Accuracy)
	OMGL (N = 77)	HGMD (N = 138)	gnomAD (N = 1,232)	Calmodulin (N = 576)
CardioBoost	**88.3*** **	**72.5*** **	**64.4*** **	**29.0*** **
M-CAP	59.7	39.9	9.8	0.3
REVEL	68.8	52.9	2.8	4.2

Supplementary Table 16. CardioBoost variant classification stratifies variants with increased disease Odds Ratio for sarcomere-encoding genes. Odd Ratios (ORs) and their confidence intervals were calculated for rare variants observed in sarcomere-encoding genes using SHaRe HCM cohorts and gnomAD. We compared the ORs for three groups of variants: (i) all rare variants, (ii) rare variants predicted disease-causing by CardioBoost (Pr ≥ 0.9, and excluding those seen in our training data), and (iii) rare variants predicted as benign by CardioBoost (Pr ≤ 0.1, and excluding those seen in our training data). The ORs of variants classified by M-CAP and REVEL were also calculated.

Gene symbol	all observed rare variants (95% CI)	CardioBoost disease-causing variants (95% CI)	CardioBoost benign variants (95% CI)	M-CAP disease-causing variants (95% CI)	M-CAP benign variants (95% CI)	REVEL disease-causing variants (95% CI)	REVEL benign variants (95% CI)
MYH7	14.5 (13.4-15.7)	14.7 (12.9-16.7)	1.2 (0.7-1.9)	14.8 (12.9-16.9)	-*	15.9 (13.1-19.2)	-*
TNNI3	12.6 (10.1-15.9)	14.0 (6.1-32.3)	3.3 (1.7-6.4)	1.0 (1 -1.1)	4.7 (1.6 – 14)	12.1 (4-35.9)	1.0 (1-1.1)
TPM1	11.2	33.7	1.4	1.0 (1 -1.1)	0.5 (0.1 – 3.6)	38.9 (5.9-256.6)	-*

	(8.2-15.3)	(18.3 – 62.2)	(0.5-3.8)				
ACTC1	11.2<br>(6.9-18.2)	15.2<br>(8.2-28.3)	1.0<br>(1-1.1)	1.0<br>(1 -1.1)	1.0<br>(1 - 1.1)	19.8<br>(9.4-42)	-*
TNNT2	6.0<br>(4.8-7.5)	17.7<br>(10.1-31.1)	2.8<br>(1.5-5.1)	1.0<br>(1 -1.1)	1.0<br>(0.1 – 7.1)	25.8<br>(3.3-199.1)	28.9<br>(5.2-161.6)
MYBPC3	5.6<br>(5.1-6.0)	55.1<br>(41-74.1)	1.2<br>(0.9-1.4)	1.0<br>(1 -1.1)	0.7<br>(0.4-1.1)	12.8<br>(7.6-21.8)	1.2<br>(0.8-1.8)
MYL2	5.2<br>(4.0-6.9)	3.8<br>(2.0-7.5)	1.0<br>(0.9-1.1)	1.0<br>(1 -1.1)	0.2<br>(0-1.6)	1.7<br>(0.4-7)	1.0<br>(1-1.1)
MYL3	2.7<br>(1.9-3.8)	7.9<br>(3.5-17.8)	0.8<br>(0.4-1.9)	1.0<br>(1 -1.1)	0.3<br>(0-2.2)	19.4<br>(8.3-45.4)	-*

*OR not calculated since the number of missense variants predicted as benign is zero in the gnomAD population.

Supplementary Table 17. Comparison of out-of-sample classification performances for alternative disease-specific classification tasks. We explored alternative variant classification models as exemplified for cardiomyopathies with relatively larger size of training data: two syndrome-specific models (HCM-specific and DCM-specific) and three gene-syndrome-specific models (*MYH7*-HCM-specific, *MYH7*-DCM-specific and *MYBPC3*-HCM-specific). Here the broadly cardiomyopathies-specific model was chosen since none of the alternative models had comparable performances.

Predictive task	Number of training variants	Precision-Recall AUC (%)
CM-specific	440	91
HCM-specific	348	79
DCM-specific	309	48
MYH7-HCM-specific	152	87
MYH7-DCM-specific	152	35
MYBPC3-HCM-specific	106	76

Supplementary Methods

The data flow diagram from data collection, machine learning model training and testing is illustrated in **Figure 1**.

Background

While we have extensively benchmarked with genome-wide tools, the idea of gene-specific or syndrome-specific models for inherited cardiac conditions have been developed previously including a MYH7-specific predictor[2], our Bayesian syndrome-specific classification predictor APPRAISE[3], a HCM-specific classification model PolyPhen-HCM[4] and a cardiomyopathy-specific model PathoPredictor[5]. Compared to these existing important works, we have improved the disease-specific classifiers in terms of the size and diversity of the predictive features and training datasets. We collected substantially relevant features (n=76) for variant classifications including conservation, existing pathogenicity scores and genetic constraint scores. Our models were trained with larger size of high-quality expert-curated variants including as many disease genes as possible (CM: genes = 16, variants = 440; IAS: genes = 7, variants = 326).

The details of data collection for training and testing are provided below.

Primary training and test data collection

We consider rare missense variants whose allele frequency is less than 0.1%, using gnomAD (v2.0.1) as our reference population. The value at 0.1% is taken as a conservative maximum credible population allele frequency[6] across a range of inherited cardiac conditions, above which variants are unlikely to cause penetrant disease. The predicted molecular consequences of variants were annotated with Ensembl Variant Effect Predictor[7] (version 91.1 for hg19/GRCh37 human genome assembly) on canonical transcripts relevant to heart tissue (**Table S1** and **Table S2**).

Pathogenic variants in sixteen genes associated with cardiomyopathies (**Table S1**) were collected from the targeted sequencing data of 9,007 patients with either HCM or DCM, recruited or referred for diagnostic sequencing at the Royal Brompton & Harefield Hospitals NHS Trust (RBH, UK), Oxford Medical Genetics Laboratories (OMGL, UK)[8], and the Partners Laboratory of Molecular Medicine (LMM, US)[9,10]. The pathogenic variants from RBH and OMGL were interpreted according to ACMG/AMP guidelines. The pathogenic variants from LMM were interpreted using equivalent previously-described clinical-grade variant classification criteria[9,10].

For inherited arrhythmia syndromes, pathogenic variants in seven genes **(Table S2)** were extracted from the ClinVar database (ClinVar Full Release 201912), considering only variants with Pathogenic or Likely pathogenic classifications and no conflicting interpretations (Benign or Likely benign).

Rare benign variants for both conditions were collected from the targeted sequencing of 2,090 healthy volunteers. The age range for the healthy volunteer cohort is 5 to 88 years (mean age = 39, SD=15). It included samples recruited from three sites: Royal Brompton Hospital (n=921, range=18-80 years, mean age=39, SD = 13), Egypt Aswan Heart Centre (n=423, range=5-79, mean age = 30, SD=10) (Aguib, Y. *et al.* Genomics of Egyptian Healthy Volunteers: The EHVol Study. *bioRxiv* (2019) https://doi.org/10.1101/680520 (unpublished data)) and Singapore National Heart Centre (n=746, range=18-88 years, mean age = 45, SD=17). These volunteers were confirmed to have no cardiac history, no family history of, or suggestive of, an inherited cardiac condition, and no evidence of cardiomyopathy or channelopathy on ECG or cardiac MRI. This cohort provides a lower disease prevalence than a general population (i.e. the prevalence of inherited cardiomyopathies and arrhythmias in a general population is estimated at ~0.75% by summing the combined prevalence of HCM, DCM, LQTS and Brugada syndrome[6]). Thus, the variants found in their disease panel genes could be considered as highly likely benign for inherited cardiac conditions, while

acknowledging the potential for a low background error rate due to incomplete and age-related penetrance.

Three genes are each associated with two related disease phenotypes in the training & test data (*MYH7* and *TNNI3* with hypertrophic and dilated cardiomyopathies; *SCN5A* with two arrhythmia syndromes, LQT & BrS), with distinct variants causing each phenotype. For each of these genes variants were aggregated so that the model was trained to discriminate disease-causing for either condition vs. benign. The phenotype associated with variation in *PLN* does not fit neatly into the clinical definitions of either HCM or DCM[1], so the output of the model for *PLN* variants is interpreted as probability of variants causing intrinsic cardiomyopathy. For all other genes the model was exposed to variants associated with just one phenotype (HCM, DCM, BrS or LQT; **Tables S1-S2**).

Additional replication test data collection

To further validate CardioBoost performance on "unseen" data, we collected additional independent data sets which did not overlap with either the training data of CardioBoost, M-CAP and REVEL or the hold-out test data of CardioBoost.

For cardiomyopathies, these pathogenic test data sets are composed of 129 Pathogenic/Likely Pathogenic variants identified in HCM patients from the SHaRe Registry[11], 15 ClinVar (ClinVar Full Release 201912)[12] variants adjudicated as Pathogenic/Likely Pathogenic for cardiomyopathies with at least two-star review status, and 145 variants of the Disease Mutation (DM) class from HGMD Pro version 201712 after excluding those also seen in HGMD version 2015.2, since these variants were used in the training of M-CAP and REVEL. For arrhythmias, 77 variants reported to be Pathogenic/Likely Pathogenic by OMGL, and 138 variants of the DM class from HGMD Pro version 201712 were collected after excluding those seen in HGMD version 2015.2. For the three calmodulin genes (*CALM1*, *CALM2* and *CALM3*), we also collected variant functional scores from a previous deep mutational scanning study[13].

In this study, a complete functional map for each possible amino acid change in calmodulin protein was generated by employing a high-throughput functional complementation assay in *S.cerevisiae*. Since the three calmodulin genes encode the same protein sequence, the functional map is same for the three genes. We think this functional map study provides an orthogonal test dataset to validate our prediction because calmodulin protein is highly conserved in eukaryotes. However, we also recognise that the yeast functional assay cannot fully indicate the clinical impact of variants specific to higher organisms[14].

We expect most variants in disease-associated genes identified in gnomAD to be benign for inherited cardiac conditions since the prevalence of inherited cardiomyopathies and arrhythmias in gnomAD should not exceed those in a general population. Since ExAC[15] variants (ExAC version release 0.3, which represents a subset of gnomAD) were used to train M-CAP and REVEL explicitly, we curated a test set of 2,003 gnomAD variants in which the variants seen in ExAC were excluded. Similarly, for arrhythmias, 1,237 gnomAD variants were collected.

Input variant features collection and pre-processing

Feature collection. We combined both variant effect features collected from previous computational tools, and original newly-derived features.

There are two types of pre-existing computational tools for prediction of variant effect: (i) those that estimate the evolutionary conservation level of the genomic site or the variant itself; (ii) those that estimate the likelihood of variant pathogenicity combing both the conservation scores and biochemical properties of a variant. We used ANNOVAR[16] to collect features from published computational tools (**Table S6**). Fourteen conservation or constraint scores of amino acid change were included from BLOSUM62[17], PAM250[17], Grantham Score[18], LRT[19], PhyloP[20], PhastCons[21], SIPHY[22], fitCons[23], GERP++[24], para_zscore[25] and misbadness (Samocha, K. E. *et al. bioRxiv* (2017). doi:10.1101/148353

(unpublished data)). To utilise the predictions of existing genome-wide tools, twenty pathogenicity scores were collected from SIFT[26], Polyphen2[27], MutationTaster[28], MutationAssessor[29], FATHMM[30], FATHMM-MKL[30], PROVEAN[31], VEST3[32], CADD[33], DANN[34], MetaSVM[35], MetaLR[35], Eigen[36], M-CAP[37], REVEL[38] and MPC (Samocha, K. E. *et al. bioRxiv* (2017). doi:10.1101/148353 (unpublished data)).

To incorporate interspecies conservation maximally, we also derived new features measuring evolutionary conservation level from orthologous sequence alignments of disease genes. Using the multiple alignment of amino acid (AA) sequences of a set of species, for a given missense variant (with known site, reference AA and alternative AA) four types of features were extracted:

$$Ratio\ of\ Reference\ AA = \frac{\text{\#orthologs in the set that have the reference AA at that site}}{\text{\#orthologs in the set that have no gap at that site}}$$

$$Ratio\ of\ Alternative\ AA = \frac{\text{\#orthologs in the set that have the alternative AA at that site}}{\text{\#orthologs in the set that have no gap at that site}}$$

$$Ratio\ of\ No-Gap = \frac{\text{\#orthologs in the set that have no gap at that site}}{\text{\#orthologs in the set}}$$

$$Ratio\ of\ Orthologs = \frac{\text{\#orthologs in the set}}{\text{\#species in the set}}$$

We downloaded multiple sequence alignments of orthologous genes from the UCSC hg19 100-way Multiz alignment[39]. The above four scores were calculated for nine different sets of species: (1) all species included in the 100-way alignment; sets of species clade: (2) Primate (3) Euarchontoglires; (4) Laurasiatheria; (5) Afrotheria; (6) Mammal; (7) Aves; (8)

Sarcopterygii and (9) Fish (For species in each clade subset see http://hgdownload.cse.ucsc.edu/goldenpath/hg19/multiz100way/).

We also derived region-level features from the AA alignment. $Mean\ Ratio\ of\ Reference\ AA$ measures the average ratio of $Ratio\ of\ Reference\ AA$ among the allele's 10 nearest neighbouring sites. Similarly, $Mean\ Ratio\ of\ No-Gap$ measures the average $Ratio\ of\ No-Gap$ among the allele's 10 nearest neighbouring sites.

Using the alignment of multiple nucleotide sequences, $Ratio\ of\ Reference\ Nucleotide$ and $Ratio\ of\ Alternative\ Nucleotide$ calculate the frequency of reference nucleotide and alternative nucleotide observed in all orthologs given there is no gap at this site respectively. Similarly, $Ratio\ of\ Reference\ Codon$ and $Ratio\ of\ Alternative\ Codon$ are derived as a measure of conservation at the codon level.

Missing features imputation. Variant pathogenicity scores derived from existing genome-wide classifiers and included as features in our model were not available for all variants considered. We estimated these missing values by using condition mean imputation. For test data, missing values were imputed by using the mean derived in the training data[40].

Features normalisation. In total, we collected 76 features per missense variant. After collecting all the features, we conducted a z-score normalisation on the features of the training data. The features in test data were also standardised using the means and standard variations of the training data.

Defining high-confidence classification performance measures

Existing machine learning variant classification tools adopted a single threshold to discriminate pathogenic and benign variants. However, the choice of this classification threshold is arbitrary

and not consistent among different tools, for example M-CAP[37] made a binary classification using a threshold with 95% true positive rate (see the relevant discussion in **Supplementary Methods**: Limitations in applying a high-sensitivity threshold for variant interpretation) and PolyPhen-2[27] made a ternary classification using two thresholds based on false positive rates.

This arbitrary choice of classification threshold might not be optimal in order to control Type I and Type II error for different applications. Moreover, the use of high-sensitivity threshold for variant classification is unlikely optimal for clinical interpretation of individual variants. Instead of using classification thresholds derived from a specific classification method/data set, here we adopt high-confidence classification definitions aligned with ACMG/AMP guideline recommendations for clinical practice[41]: the classification of variants into Likely Pathogenic/Pathogenic or Likely Benign/Benign is proposed to be with at least 90% classification certainty. In other words, variants with pathogenicity score equal to or larger than 0.9 would be classified as “disease-causing” and those with pathogenicity score equal to or smaller than 0.1 are classified as “benign”. Variants with pathogenicity scores between 0.1 and 0.9 receive an indeterminate classification (variants of unknown significance) (**Figure 1b** and **Figure 1c**).

With the defined high-certainty classification thresholds, we derive the corresponding confusion matrix (**Figure 1c**) from which a series of measures of direct clinical relevance can be computed. We use TPR, the proportion of actual pathogenic variants predicted to be disease-causing, and PPV, the proportion of predicted disease-causing variants that are correctly classified, to evaluate the classifier’s ability to classify pathogenic variants. TNR, the proportion of actual benign variants predicted to be benign and NPV, the proportion of predicted benign variants that are correctly classified are used to assess benign classifications correspondingly. Taking both cases together, the accuracy of high-confidence classifications measures the probability that a classification in the actionable range is correct. The proportion of clinically indeterminate classifications measures the probability of a variant not classified

with clinical confidence. Formulae for each measure of clinical relevance we used are described in the below session.

Calculation of high-confidence classification measures

	Predicted disease-causing	Predicted benign	Indeterminate	
Actual pathogenic	TP	FN		T
Actual benign	FP	TN		F
	P	N		

Based on the confusion matrix shown in **Figure 1c (shown above)**, we calculated the following ratios of clinical relevance in variant interpretation given *n* test variants

$$\mathrm{TPR} = \frac{\mathrm{TP}}{\mathrm{T}}$$

$$\mathrm{TNR} = \frac{\mathrm{TN}}{\mathrm{F}}$$

$$\mathrm{FPR} = \frac{\mathrm{FP}}{\mathrm{F}}$$

$$\mathrm{PPV} = \frac{\mathrm{TP}}{\mathrm{P}}$$

$$\mathrm{NPV} = \frac{\mathrm{TN}}{\mathrm{N}}$$

$$\mathrm{FNR} = \frac{\mathrm{FN}}{\mathrm{T}}$$

$$\text{Number of high} - \text{confidence classifications} = \text{P} + \text{N}$$

$$\text{Number of indeterminate classifications} = n - (\text{P} + \text{N})$$

$$\text{Proportion of high} - \text{confidence classifications} = \frac{\text{P} + \text{N}}{n}$$

$$\text{Accuracy of high} - \text{confidence classifications} = \frac{\text{TP} + \text{TN}}{\text{P} + \text{N}}$$

$$\text{Overall accuracy} = \frac{\text{TP} + \text{TN}}{n}$$

$$\text{Proportion of indeterminate classifications} = \frac{n - (\text{P} + \text{N})}{n},$$

where T: Actual pathogenic, F: Actual benign, P: Predicted disease-causing (Pathogenicity $Pr \geq 0.9$), N: Predicted benign ($Pr \leq 0.1$), Indeterminate: $0.1 < Pr < 0.9$, TP: True Positive, TN: True Negative, FP: False Positive, FN: False Negative, T = TP + FN, F = FP + TN, P = TP + FP and N = FN + TN.

Machine learning model training and selection

The analyses were conducted using the R environment[23] and the package mlr[24]. We trained and tested representatives of each of the major classes of statistical and machine learning methods in order to obtain the best classification performances over our training data. Neural network methods were not included due to limited scope for interrogation and interpretation of feature weightings. Classification algorithms included in the analysis are: Classification and Regression Tree (CART)[44], K nearest neighbours (KNN)[45], Elastic Net Logistic Regression (GLMNET)[46], Support Vector Machine with Radial Basis Kernel Function (SVM-RBF)[47],

Random Forest (RF)[48], Bayesian Additive Regression Trees (BART)[49], Adaptive Boosting (AdaBoost)[47], Gradient Boosting Tree (GBM)[47] and Extreme Gradient Boosting (XGBoost)[50].

To fine-tune hyper parameters for each model and identify the model with the best generalisation performance (i.e. best prediction performance on “unseen” data), we applied a nested cross-validation[51]. In this nested cross-validation, the inner-test set (also called “validation set” or “development set”) is used to choose the optimal set of hyperparameter for a given classification algorithm. After the classification algorithm is fitted on the inner loop data set, the outer test set is used to select the best tuned classification algorithm with respect to its performance on “unseen” test data. We used 5-fold cross-validation in the inner cross-validation loop and 10-fold in the outer cross-validation loop.

The selection of the best classification algorithm is not trivial. To this end, we pre-specified the following optimisation goals:

Goal 1: The optimal classifier outperforms genome-wide machine learning variant classification tools on overall classification measured using PR-AUC.

We consider the PR-AUC as a conventional threshold-independent performance measure. In the training process, PR-AUC is chosen as the objective measure in the inner loop for hyperparameter tuning, i.e. for each candidate classification algorithm considered, the hyperparameters that yield the highest PR-AUC are selected. Then the classification performance of each optimised algorithm is assessed using the outer CV loop.

Goal 2: The optimal classifier has the best Matthews Correlation Coefficient[52] (MCC) using the defined 90% high-confidence classification threshold.

Our aim is to find the optimal classifier that balances both Type I and Type II errors at the 90% high-confidence classification thresholds. When we apply the defined high-confidence classification above, variants are classified into one of three categories: disease-causing, benign and indeterminate. Since the most common application of a genetic diagnosis in cardiogenetic practice is familial evaluation and predictive testing, where management of negative and inconclusive genetic test results are equivalent[53], we group these variants together for the purposes of model selection, and focus on performance at the higher actionable threshold, comparing disease-causing versus non-actionable indeterminate/benign/likely benign.

We use the MCC, a measure of the correlation between observed and predicted binary classifications that is relatively robust in an imbalanced data set[54], defined as:

$$MCC = \frac{\mathrm{TP} \times \mathrm{TN} - \mathrm{FP} \times \mathrm{FN}}{\sqrt{(\mathrm{TP} + \mathrm{FP})(\mathrm{TP} + \mathrm{FN})(\mathrm{TN} + \mathrm{FP})(\mathrm{TN} + \mathrm{FN})}}.$$

A higher MCC reflects a stronger correlation between observed and predicted binary classification, indicative of performance at the ≥ 0.9 threshold most relevant in this context. Ideally, we would like to select a classifier that performs best on both goals. If there is more than one classifier satisfying both Goal 1 and Goal 2, we pre-specify selection of the models using Goal 2, given the most immediate relevance to this task.

The performance of each candidate machine learning algorithm and the representative benchmarking genome-wide variant classification tools (M-CAP and REVEL) in the nested cross-validation are shown in **Table S7** and **Table S8**. For cardiomyopathy variants, as shown in **Table S7** the candidate algorithms that outperform M-CAP and REVEL on all standard classification measures to meet Goal 1 were GLMNET, CART, RF, BART, XGBoost, GBM, AdaBoost, KNN and SVM. Since AdaBoost had the highest MCC score to meet Goal 2, it was

selected as the best model. Next the best hyperparameter set for AdaBoost ("loss=exponential" and "nu=0.207") was selected using 5-fold cross validation on the whole cardiomyopathy variant training set. The selected model was trained on the whole training set to generate predictions on unseen data.

Similarly, for inherited arrhythmia syndrome variants, AdaBoost was selected as the best-performing candidate (hyperparameters "loss=exponential" and "nu=0.435"). The prediction model was then trained using the whole arrhythmia training set.

Permutation significance test

Given a performance measure, we used one-sided permutation test[55] to test whether an observed performance measure of one classifier was significantly better than that of the other classifier. The null hypothesis is that the two classifiers perform the same on this measure. The null distribution is estimated by randomly exchanging observations between the classifiers 10,000 times. Here, an observation represents a variant pathogenic probability predicted by a classifier. *P*-value is estimated as the number of times the permutated difference is larger than the observed difference.

Replication without reliance on gold-standard

To ensure robustness to misclassification in the "gold-standard" out-of-sample test data, we employed two orthogonal approaches to assess CardioBoost's discrimination of pathogenic variants and benign variants. First, we compared the proportion of rare variants in individuals with and without disease, and stratified these variants using CardioBoost. We derived the odds ratio (OR), which provides an estimate of gene-disease association.

Second, we compared the survival outcomes of individuals with HCM, stratified by genotypes classified by CardioBoost. We applied CardioBoost to variants found in a cohort of 803 patients with HCM and a rare missense variant in one of eight HCM-associated genes, and

compared survival with 1,927 genotype-negative HCM patients. We did not consider individuals carrying variants seen in our training data set. The “event-free survival” time (i.e. time until first major adverse clinical event) was analysed using Kaplan-Meier survival analysis and the Cox hazard-regression model.

Survival analysis

We collected genotype and clinical outcome data for patients with cardiomyopathy from the SHaRe HCM registry (data release 2019Q3).

We included patients with a diagnosis of HCM, at least 1 clinic visit and at least 1 assessment of left ventricular wall thickness, and only one missense variant in any of eight genes encoding sarcomere proteins (*MYBPC3*, *MYH7*, *TNNT2*, *TNNI3*, *TPM1*, *MYL2*, *MYL3*, and *ACTC1*). Variants jdentified in SHaRe were classified by SHaRe experts according to ACMG/AMP guidelines. Patients with potentially pathogenic variants in genes encoding non-sarcomere proteins (i.e. HCM genocopies) were excluded.

The primary outcome measure was a composite comprising the first occurrence of: sudden cardiac death, resuscitated cardiac arrest, appropriate implantable cardioverter-defibrillator therapy, cardiac transplantation, left ventricular assist device implantation, New York Heart Association class III-IV symptoms, all-cause mortality, atrial fibrillation, stroke, or death, as previously described[11].

Patients were censored either at date of first event, or at last follow-up clinical visit if event-free.

Data leakage does not explain the superior performance of CardioBoost compared with existing tools

Since CardioBoost training and test data may contain variants used as training data for published genome-wide classification tools whose pathogenicity scores were used as input features by CardioBoost, we also assessed whether using indirectly “seen" data would make CardioBoost overfit and outcompete existing genome-wide classifiers. In particular, we considered previously “seen” variants used in training M-CAP and REVEL. M-CAP was trained on variants of Disease Mutation (DM) Class from HGMD version 2015.2 and ExAC. REVEL was trained on variants of DMs from HGMD version 2015.2 and the Exome Sequencing Project (ESP), the Atherosclerosis Risk in Communities (ARIC) study and the 1000 Genomes Project (KGP) (ESP and KGP are contributing projects in ExAC). We extracted a set of “seen” variants from CardioBoost training data if they are ever seen in the DM Class of HGMD version 2015.2 and ExAC. The remaining variants in the training data constitute the set of purely “unseen” data. We investigated the impact of using “seen” data from two different viewpoints. One is whether “seen” variants have the same classification as those in our training data. In Cardiomyopathies 323 out of 440 training variants were seen before in HGMD or ExAC. For the DM variants reported in HGMD before, 53 out of 206 cases have an opposite classification as in our training data. In Arrhythmias, there are 253 out of 308 variants ever seen in HGMD or ExAC. Among the 170 DM variants reported in HGMD previously, 38 of them have opposite classification in our training data. This suggests that even if some variants were used in building previously genome-wide classifiers, their classifications are not necessarily correct and thus it makes the prediction tools less accurate. The second aspect is to assess whether our machine learning tool could still outcompete M-CAP and REVEL on completely “unseen” data. We compared the prediction performance of stratified hold-out test sets: purely “unseen” data and “seen” data (see **Table S10**) with the unstratified hold-out test set. The accuracy was used as an overall measure to compare the performance of each dataset. For cardiomyopathies and arrhythmias, the performances of three datasets were comparable and not significantly different. Overall, we found out the variants used in previous genome-wide tools were not necessarily accurately classified. Our machine learning tool did improve on

cardiomyopathy- and arrhythmia-specific prediction both on “seen” and “unseen data” by leveraging over multiple diverse computational pieces of evidence.

Limitations in applying a high-sensitivity threshold for variant interpretation

In M-CAP, the authors defined a single low pathogenicity threshold as clinically relevant to predict disease-causing variants such that M-CAP could have 95% expected true positive rate (sensitivity). Given a data set, while using a low single classification threshold to increase TPR will decrease the number of false negative predictions, the binary classifier would tend to increase the number of false positive predictions (i.e., truly benign variants predicted to be disease-causing) as well. An ideal classification threshold would be the one that minimize the total sum of the cost of both errors. While one might prioritise sensitivity for variant prioritisation in some contexts, in the context of clinical variant interpretation, we suggest that the cost of a false positive prediction is at least equivalent to, and in most situations higher than, the cost of a false negative prediction. In neglecting to control the Type II error to have high true positive rate, there would be two negative consequences: (i) Low positive predictive value: this could be demonstrated as the negative correlation between the true positive rate and positive predictive value using the Precision-Recall Curve (**Figure 2a** and **Figure 2c**); (ii) High false positive rate: this is demonstrated as the positive correlation between the true positive rate and false positive rate (i.e., 1-TNR) (**Figure 2b** and **Figure 2d**). Even though the ACMG guidelines recommend not to use one computational tool as a sole evidence, but to consider the concordance of multiple computational tools for variant interpretation, the application of a computational tool of high TPR but low TNR or high FPR along with other computational tools would still make the clinical interpretation process rather difficult. For example, the disease-causing prediction of a computation tool for a truly benign variant is very likely to conflict with the correct prediction from the other computational tools or the other lines of evidence of pathogenicity. The contradictory evidence would increase the likelihood that the variant is classified as variant of uncertain significance (VUS).

Calibration of PPV and NPV

Given a new dataset or testing context, we could estimate the PPV and NPV of a classifier given the proportion of pathogenic variants amongst variants undergoing classification (Variant Proportion):

$$\text{Variant Proportion} = \frac{\text{Number of pathogenic variants}}{\text{Number of pathogenic variants} + \text{Number of benign variants}}$$

$$\text{PPV} = \frac{\text{TPR} \times \text{Variant Proportion}}{\text{TPR} \times \text{Variant Proportion} + \text{FPR} \times (1 - \text{Variant Proportion})}$$

$$\text{NPV} = \frac{\text{TNR} \times (1 - \text{Variant Proportion})}{\text{TNR} \times (1 - \text{Variant Proportion}) + \text{FNR} \times \text{Variant Proportion}}$$

where TPR: True Positive Rate and TNR: True Negative Rate as defined in (1) and (2), respectively.

Estimating the proportion of pathogenic missense variants in a diagnostic series and a general population

In order to estimate the PPV and NPV when applying CardioBoost in a diagnostic series and a general population, we first estimate the proportion of pathogenic missense variants of these two populations.

Since in variant interpretation, the limitation of false positive prediction is prioritised. Here we want to derive a reasonably conservative estimate of PPVs by assuming that pathogenic missense variants are penetrant and that the burden of rare missense variants in controls provides an estimate of the burden of rare benign missense variants in any population either cases or control. These assumptions would provide the lower bound of the proportion of pathogenic variants, which is the lower bound of PPV based on.

Based on the above assumptions, the proportion of rare pathogenic missense variants, for a given gene or a gene set, amongst variants identified in a group of patients with disorders could be approximated as:

$$\text{Variant proportion in a case series} = \frac{\text{Burden of pathogenic variants in cases}}{\text{Burden of rare variants in cases}}$$

$$\text{Burden of pathogenic variants in cases} = \text{Burden of rare variants in cases} - \text{Burden of rare variants in control}$$

Similarly, the proportion of rare pathogenic missense variants in a general population could be approximated as:

$$\text{Variant proportion in a general population} = \frac{\text{Burden of pathogenic variants in a general population}}{\text{Burden of rare variants in a general population}}$$

$$\text{Burden of rare variants in a general population} = \text{Burden of pathogenic variants in a general population} + \text{Burden of benign variants in a general population}$$

$$\text{Bruden of pathogenic variants in a general population} = \text{Prevalence of disease} \times \text{Burden of pathogenic variants in cases} = \text{Prevalence of disease} \times (\text{Burden of rare variants in cases} - \text{Burden of rare variants in control})$$

$$\text{Burden of benign variants in a general population} = \text{Burden of rare variants in control}$$

For cardiomyopathies, here we consider both dilated cardiomyopathy (DCM) and hypertrophic cardiomyopathy (HCM). The disease prevalence for DCM is estimated as 1/250 and 1/500 for HCM[56]. Thus, adding the prevalence of two conditions, the disease prevalence for cardiomyopathies is

$$\frac{1}{250} + \frac{1}{500} \approx 0.006$$

Using cohort studies from OMGL and LMM[8], the burden of rare missense variants in cases is estimated at 27%. *PTPN11* (it was not sequenced in these cohorts and its contribution to cases is assumed to be marginal) was excluded in the analysis here. Using gnomAD[15] reference population as control, the burden of rare missense variants in control was estimated to be 11% by adding the allele frequencies of rare missense variants seen in gnomAD for all cardiomyopathies-related genes (excluding *PTPN11*).

Thus, the proportion of rare missense variants pathogenic to cardiomyopathies in a diagnostic series is estimated with ~ 60%. The proportion of rare missense variants pathogenic to cardiomyopathies in a general population is estimated as ~1%.

Likewise, the proportions of rare missense variants pathogenic to arrhythmias in a diagnostic series and in a general population are estimated as ~71% and ~0.4% respectively. The disease prevalence of arrhythmias in a general population is ~0.2% by adding the disease prevalence of Long QT syndrome (1/2000) and Brugada syndrome (1/1000). Since the arrhythmias-related genes are not widely assessed in large LQTS and Brugada cohort studies[57,58], here we could only consider four arrhythmias-associated genes *KCNE1, KCNH2*, *KCNQ1* and *SCN5A* here from the LQTS and Brugada cohort studies[57,58], which provides us a lower bound of exact variant proportion. The burden of rare missense variants in arrhythmias

is estimated as 18%. From gnomAD database, we could estimate the burden of rare missense variants in control (only including *KCNE1, KCNH2*, *KCNQ1* and *SCN5A*) as 5%.

References

1. Ingles, J. *et al.* Evaluating the Clinical Validity of Hypertrophic Cardiomyopathy Genes. *Circ. Genomic Precis. Med.* **12**, (2019).
2. Al-Numair, N. S. *et al.* The structural effects of mutations can aid in differential phenotype prediction of beta-myosin heavy chain (Myosin-7) missense variants. *Bioinformatics* (2016). doi:10.1093/bioinformatics/btw362
3. Ruklisa, D., Ware, J. S., Walsh, R., Balding, D. J. & Cook, S. A. Bayesian models for syndrome- and gene-specific probabilities of novel variant pathogenicity. *Genome Med.* **7**, 5 (2015).
4. Jordan, D. M. *et al.* Development and validation of a computational method for assessment of missense variants in hypertrophic cardiomyopathy. *Am. J. Hum. Genet.* (2011). doi:10.1016/j.ajhg.2011.01.011
5. Evans, P. *et al.* Genetic variant pathogenicity prediction trained using disease-specific clinical sequencing data sets. *Genome Res.* (2019). doi:10.1101/gr.240994.118
6. Whiffin, N. *et al.* Using high-resolution variant frequencies to empower clinical genome interpretation. *Genet. Med.* **19**, 1151–1158 (2017).
7. McLaren, W. *et al.* The Ensembl Variant Effect Predictor. *Genome Biol.* **17**, (2016).
8. Walsh, R. *et al.* Reassessment of Mendelian gene pathogenicity using 7,855 cardiomyopathy cases and 60,706 reference samples. *Genet Med* **19**, 192–203 (2017).
9. Pugh, T. J. *et al.* The landscape of genetic variation in dilated cardiomyopathy as surveyed by clinical DNA sequencing. *Genet. Med.* **16**, 601–608 (2014).
10. Alfares, A. A. *et al.* Results of clinical genetic testing of 2,912 probands with hypertrophic cardiomyopathy: Expanded panels offer limited additional sensitivity. *Genet. Med.* **17**, 880–888 (2015).

11. Ho, C. Y. *et al.* Genotype and lifetime burden of disease in hypertrophic cardiomyopathy: insights from the Sarcomeric Human Cardiomyopathy Registry (SHaRe). *Circulation* **138**, 1387–1398 (2018).

12. Landrum, M. J. *et al.* ClinVar: improving access to variant interpretations and supporting evidence. *Nucleic Acids Res.* **46**, D1062–D1067 (2018).

13. Weile, J. *et al.* A framework for exhaustively mapping functional missense variants. *Mol. Syst. Biol.* (2017). doi:10.15252/msb.20177908

14. Zhang, J. *et al.* Assessing predictions on fitness effects of missense variants in calmodulin. *Hum. Mutat.* (2019). doi:10.1002/humu.23857

15. Lek, M. *et al.* Analysis of protein-coding genetic variation in 60,706 humans. *Nature* **536**, 285–291 (2016).

16. Wang, K., Li, M. & Hakonarson, H. ANNOVAR: functional annotation of genetic variants from high-throughput sequencing data. *Nucleic Acids Res.* **38**, e164–e164 (2010).

17. Henikoff, S. & Henikoff, J. G. Amino acid substitution matrices from protein blocks. *Proc. Natl. Acad. Sci.* **89**, 10915–10919 (1992).

18. Grantham, R. Amino Acid Difference Formula to Help Explain Protein Evolution. *Science.* **185**, 862–864 (1974).

19. Chun, S. & Fay, J. C. Identification of deleterious mutations within three human genomes. *Genome Res.* **19**, 1553–1561 (2009).

20. Siepel, A., Pollard, K. S. & Haussler, D. *New Methods for Detecting Lineage-Specific Selection*. *Research in Computational Molecular Biology* (Springer Berlin Heidelberg, 2006).

21. Siepel, A. *et al.* Evolutionarily conserved elements in vertebrate, insect, worm, and yeast genomes. *Genome Res.* **15**, 1034–1050 (2005).

22. Garber, M. *et al.* Identifying novel constrained elements by exploiting biased substitution patterns. *Bioinformatics* **25**, (2009).

23. Gulko, B., Hubisz, M. J., Gronau, I. & Siepel, A. A method for calculating probabilities

of fitness consequences for point mutations across the human genome. *Nat. Genet.* **47**, 276–283 (2015).

24. Davydov, E. V. *et al.* Identifying a high fraction of the human genome to be under selective constraint using GERP++. *PLoS Comput. Biol.* **6**, (2010).

25. Lal, D. *et al.* Gene family information facilitates variant interpretation and identification of disease-associated genes in neurodevelopmental disorders. *Genome Med.* (2020). doi:10.1186/s13073-020-00725-6

26. Kumar, P., Henikoff, S. & Ng, P. C. Predicting the effects of coding non-synonymous variants on protein function using the SIFT algorithm. *Nat. Protoc.* **4**, 1073–1082 (2009).

27. Adzhubei, I., Jordan, D. M. & Sunyaev, S. R. Predicting functional effect of human missense mutations using PolyPhen-2. *Curr. Protoc. Hum. Genet.* **76**, 7.20.1-7.20.41 (2013).

28. Schwarz, J. M., Cooper, D. N., Schuelke, M. & Seelow, D. Mutationtaster2: Mutation prediction for the deep-sequencing age. *Nature Methods* **11**, 361–362 (2014).

29. Reva, B., Antipin, Y. & Sander, C. Predicting the functional impact of protein mutations: Application to cancer genomics. *Nucleic Acids Res.* **39**, (2011).

30. Shihab, H. A. *et al.* Ranking non-synonymous single nucleotide polymorphisms based on disease concepts. *Hum. Genomics* **8**, (2014).

31. Choi, Y., Sims, G. E., Murphy, S., Miller, J. R. & Chan, A. P. Predicting the Functional Effect of Amino Acid Substitutions and Indels. *PLoS One* **7**, (2012).

32. Carter, H., Douville, C., Stenson, P. D., Cooper, D. N. & Karchin, R. Identifying Mendelian disease genes with the Variant Effect Scoring Tool. *BMC Genomics* **14**, S3 (2013).

33. Kircher, M. *et al.* A general framework for estimating the relative pathogenicity of human genetic variants. *Nat. Genet.* **46**, 310–315 (2014).

34. Quang, D., Chen, Y. & Xie, X. DANN: a deep learning approach for annotating the pathogenicity of genetic variants. *Bioinformatics* **31**, 761 (2014).

35. Dong, C. *et al.* Comparison and integration of deleteriousness prediction methods for nonsynonymous SNVs in whole exome sequencing studies. *Hum. Mol. Genet.* **24**, 2125–2137 (2015).
36. Ionita-Laza, I., McCallum, K., Xu, B. & Buxbaum, J. D. A spectral approach integrating functional genomic annotations for coding and noncoding variants. *Nat Genet* **48**, 214–220 (2016).
37. Jagadeesh, K. A. *et al.* M-CAP eliminates a majority of variants of uncertain significance in clinical exomes at high sensitivity. *Nat Genet* **48**, 1581–1586 (2016).
38. Ioannidis, N. M. *et al.* REVEL: An Ensemble Method for Predicting the Pathogenicity of Rare Missense Variants. *Am. J. Hum. Genet.* **99**, 877–885 (2016).
39. Kuhn, R. M., Haussler, D. & Kent, W. J. The UCSC genome browser and associated tools. *Brief. Bioinform.* **14**, 144–161 (2013).
40. Schafer, J. L. & Graham, J. W. Missing data: Our view of the state of the art. *Psychol. Methods* **7**, 147–177 (2002).
41. Richards, S. *et al.* Standards and guidelines for the interpretation of sequence variants: a joint consensus recommendation of the American College of Medical Genetics and Genomics and the Association for Molecular Pathology. *Genet Med* **17**, 405–423 (2015).
42. R Core Team. R: A Language and Environment for Statistical Computing. (2017).
43. Bischl, B. *et al.* mlr: Machine Learning in R. *J. Mach. Learn. Res.* **17**, 1–5 (2016).
44. Breiman, L., Friedman, J. H., Olshen, R. A. & Stone, C. J. *Classification and Regression Trees*. (Taylor & Francis, 1984).
45. Cover, T. & Hart, P. Nearest neighbor pattern classification. *IEEE Trans. Inf. Theory* **13**, 21–27 (1967).
46. Zou, H. & Hastie, T. Regularization and variable selection via the elastic-net. *J. R. Stat. Soc.* **67**, 301–320 (2005).
47. Hastie, T., Tibshirani, R. & Friedman, J. *The elements of statistical learning: data mining, inference and prediction*. (Springer, 2009).

48. Breiman, L. Random forests. *Mach. Learn.* **45**, 5–32 (2001).
49. Chipman, H. A., George, E. I. & McCulloch, R. E. BART: Bayesian additive regression trees. *Ann. Appl. Stat.* **6**, 266–298 (2012).
50. Chen, T. & Guestrin, C. *XGBoost : Reliable Large-scale Tree Boosting System*. *Proceedings of the 22nd ACM SIGKDD International Conference on Knowledge Discovery and Data Mining* (ACM, 2016).
51. Cawley, G. C. & Talbot, N. L. C. On Over-fitting in Model Selection and Subsequent Selection Bias in Performance Evaluation. *J. Mach. Learn. Res.* (2010).
52. Boughorbel, S., Jarray, F. & El-Anbari, M. Optimal classifier for imbalanced data using Matthews Correlation Coefficient metric. *PLoS One* **12**, (2017).
53. Cirino, A. L. *et al.* Role of genetic testing in inherited cardiovascular disease: A review. *JAMA Cardiol.* **2**, 1153–1160 (2017).
54. Chicco, D. Ten quick tips for machine learning in computational biology. *BioData Min.* **10**, (2017).
55. Good, P. I. *Resampling methods : a practical guide to data analysis*. (Birkhäuser, 2010).
56. Hershberger, R. E., Hedges, D. J. & Morales, A. Dilated cardiomyopathy: The complexity of a diverse genetic architecture. *Nature Reviews Cardiology* **10**, 531–547 (2013).
57. Kapplinger, J. D. *et al.* Spectrum and prevalence of mutations from the first 2,500 consecutive unrelated patients referred for the FAMILION long QT syndrome genetic test. *Hear. Rhythm* **6**, 1297–1303 (2009).
58. Kapplinger, J. D. *et al.* An international compendium of mutations in the SCN5A-encoded cardiac sodium channel in patients referred for Brugada syndrome genetic testing. *Hear. Rhythm* **7**, 33–46 (2010).
